# Distractor processing of emotional faces: behavioral and women-only fMRI findings on the perceptual load–dilution debate

**DOI:** 10.3389/fnhum.2026.1963554

**Published:** 2026-09-21

**Authors:** Özlem Ertan, Zeynel Baran, Metehan Çiçek

**Affiliations:** 1 Department of Psychology, Ankara Medipol University, Ankara, Türkiye; 2 Neuroscience and Neurotechnology Center of Excellence, Ankara, Türkiye; 3 Department of Psychology, Hacettepe University, Ankara, Türkiye; 4 Department of Physiology, School of Medicine, Ankara Medipol University, Ankara, Türkiye

**Keywords:** dilution, distractor interference, emotional attention, fMRI, perceptual load, selective attention

## Abstract

**Introduction:**

The perceptual load–dilution debate asks whether reduced distractor interference under demanding visual conditions reflects limited perceptual capacity or competition from additional neutral items. Because this debate has relied largely on neutral stimuli, it remains unclear whether the same mechanisms govern emotionally meaningful distraction. We examined this question using an all-faces visual search paradigm that separated cue-based attentional demand from display composition while minimizing target–distractor category confounds.

**Methods:**

In Experiment 1, 60 participants completed a face-discrimination task manipulating cue size, face size, target emotion (happy, angry), and distractor type (neutral, congruent, incongruent). In Experiment 2, 30 healthy right-handed women completed an fMRI-adapted version of the task during 3T scanning.

**Results:**

Behaviorally, distractor interference increased with cue size selectively for happy targets, yielding a reversed load effect, whereas face size did not alter interference. During scanning, congruency scores were not significantly modulated by cue size, face size, or target emotion. Neurally, incongruent trials increased activity across bilateral amygdala, anterior cingulate cortex, dorsolateral prefrontal cortex, and frontal eye fields, and additionally engaged visual, parietal, subcortical, and motor regions. Cue size and face size showed different neural profiles, while emotion- and congruency-related responses depended on the broader task configuration. Left dorsolateral prefrontal congruency effects varied jointly with cue size and target emotion, whereas emotion-related responses in left anterior cingulate cortex and right amygdala depended on the cue-by-face configuration.

**Discussion:**

These findings do not support a dilution-specific amygdala mechanism. Instead, they indicate that cue-based attentional demand and display composition make different but interacting contributions to emotional distractor processing, and that neural sensitivity to distraction can remain evident when its behavioral expression is weak.

## 1 Introduction

Selective attention research has long debated the mechanisms of distractor processing. Under the dominant framework, the perceptual load (PL) theory (Lavie, 1995; Lavie and Tsal, 1994), how much task-irrelevant information is processed depends on available perceptual capacity. High load (HL) exhausts capacity on task-relevant processing and reduces distractor interference, whereas low load (LL) leaves capacity that spills over onto irrelevant stimuli, increasing distractor processing. Load is typically manipulated via the number and heterogeneity of display stimuli: homogeneous non-targets represent LL, heterogeneous ones HL (for a review, see Ertan-Kaya, 2022). Lavie (1995, 2005) argues that the load is the key determinant of efficient attentional selection.

The dilution account (DA; Benoni and Tsal, 2010; Tsal and Benoni, 2010) instead attributes reduced interference to neutral display items competing with the distractor at the representational level and diluting it, rather than to capacity limits (see the Stroop dilution effect; Kahneman and Chajczyk, 1983). Thus, manipulating load by adding neutral objects can confound load with dilution. Indeed, holding the number of neutral objects constant while varying task difficulty can produce a “reversed load effect,” in which higher task demand increases distractor interference (see Benoni, 2025).

Chen and Cave (2013), while supporting dilution, proposed that attentional zoom rather than stimulus number is decisive: what matters is which stimuli fall within the attentional focus. Their pre-cueing method, together with related work by Wilson et al. (2011), separates cue-based target-location uncertainty from the number of items in the display. Increasing the number of possible target locations broadens the spatial selection problem without necessarily increasing display density, whereas adding neutral items changes the display composition without altering target-location uncertainty. This distinction is especially useful for evaluating whether load and dilution are behaviorally and neurally interchangeable mechanisms. Consistent with this broader view, computational accounts have proposed that apparent load effects can emerge from interactions among stimulus salience, competitive representations, and top-down attentional biases rather than from perceptual capacity alone (Neokleous et al., 2016).

The load–dilution debate has relied predominantly on neutral letters and simple shapes, leaving open the question of how these mechanisms operate with emotionally meaningful stimuli. Emotional information can receive prioritized processing and interact strongly with attentional selection (Pessoa, 2005; Vuilleumier, 2005). Facial expressions are particularly informative because face perception and attention are tightly coupled (Palermo and Rhodes, 2007), yet the efficiency of selecting angry versus happy expressions varies with stimulus composition, perceptual distinctiveness, and task demands (Calvo and Marrero, 2009; Horstmann et al., 2012; Savage et al., 2016). Emotional faces, therefore, provide a stringent test of whether cue-based demand and dilution generalize beyond neutral displays.

Existing studies combining perceptual load with emotional faces generally use one of two approaches. Some vary the number of faces while observers search for an emotional target among neutral faces (Krysko and Rutherford, 2009; Savage et al., 2016), which addresses search efficiency but does not isolate interference from a spatially distinct distractor. Moreover, emotional-face search performance is sensitive to task configuration, including target expectancy and the perceptual characteristics of the stimulus set (Williams et al., 2005; Yiend, 2010). Others present emotional faces as distractors while participants perform a task on letters or another stimulus category (Gupta et al., 2016; Gupta and Wentura, 2025; Yates et al., 2010). In such designs, category differences between target and distractor can be intertwined with perceptual load, salience, and emotional relevance. An all-faces design in which target, neutral fillers, and distractor belong to the same broad stimulus category, therefore offers a cleaner test of the load–dilution distinction.

Neural evidence likewise suggests that emotional distraction is shaped by both stimulus significance and attentional context. Emotional facial expressions engage the amygdala and associated affective systems (Whalen et al., 2001), while increasing attentional demands can recruit frontoparietal control regions, including the anterior cingulate cortex (ACC), dorsolateral prefrontal cortex (DLPFC), and frontal eye fields (FEF) (e.g., Bishop et al., 2007; Wei et al., 2013). However, amygdala (AMG) responses to emotional distractors are not uniformly abolished by high demand: their persistence depends on factors such as anxiety, broader individual differences in affective functioning, available capacity, and the attentional relevance of the emotional stimulus (Bishop et al., 2007; Cornwell et al., 2011; Silvert et al., 2007). Depressive symptoms are also relevant to the present paradigm because they have been associated with broader deficits in inhibitory control (Yitzhak et al., 2023), as well as impaired inhibition of task-irrelevant emotional information and increased interference from negative affective content (Shimony et al., 2021). More broadly, this competition for processing resources between emotionally significant stimuli and ongoing task demands is not specific to face perception: comparable competitive dynamics have been observed with emotional pictures embedded in unrelated foreground tasks (Müller et al., 2008) and across cognitive tasks more broadly using combined EEG and eye-tracking measures (Mora-Quiroga et al., 2025). Indeed, facial expressions and complex affective scenes engage substantially overlapping neural networks, including the amygdala and ventromedial prefrontal cortex, alongside some format-specific differences (Britton et al., 2006), suggesting that stimulus arousal and valence modulate attention through shared, domain-general mechanisms rather than face-specific pathways. In contrast to the extensive behavioral literature on dilution, the neural consequences of manipulating dilution independently of cue-based demand remain poorly characterized. This leaves unresolved whether load and dilution converge on the same control systems or alter emotional distractor processing through partly different task-dependent patterns.

Two unresolved issues, therefore, remain. First, most load–dilution studies cannot determine whether conclusions derived from letters and simple shapes generalize to emotionally meaningful distractors whose processing is itself attention sensitive. Second, behavioral similarity or divergence between cue-based demand and display dilution does not establish whether these manipulations recruit overlapping neural systems. Separating target-location uncertainty from display composition within a single stimulus category, therefore, provides a stringent test of whether the two manipulations are functionally interchangeable. Combining this design with fMRI further allows behavioral interference and neural sensitivity to distraction to be evaluated independently.

The present study examined whether cue-based perceptual demand and display-based dilution make similar or different contributions to emotional distractor processing once target–distractor category confounds are minimized. To our knowledge, it is among the first studies to test the dilution account with emotional stimuli in a controlled all-faces design and to combine this manipulation with fMRI. Happy and angry faces were selected to be comparable in arousal and salience, allowing target emotion to be examined without treating negative valence as the only emotionally relevant condition. This design also provides a bridge between basic selective-attention theory and human cognitive neuroscience, where altered attentional control and emotion–attention interactions are implicated across neuropsychiatric conditions (Bishop, 2007; Cisler and Koster, 2010).

We predicted robust interference from incongruent relative to congruent distractors and tested whether its magnitude would be more strongly influenced by display dilution than by cue-based perceptual demand. Because both anger and happiness advantages have been reported in visual search (Calvo and Marrero, 2009; Krysko and Rutherford, 2009; Pitica et al., 2012; Savage et al., 2016), target emotion was examined as a moderator rather than assigned a single directional prediction. At the neural level, we expected distractor conflict and increasing task demands to engage frontal attentional control regions (ACC, DLPFC, FEF), whereas emotional processing was expected to involve the AMG. Whole-brain analyses were used to characterize additional visual, parietal, and subcortical systems recruited by these manipulations.

## 2 Materials and methods

### 2.1 Experiment 1: behavioral study

#### 2.1.1 Participants

Seventy-one volunteer undergraduates from Hacettepe University participated in Experiment 1. Because of potential effects on emotional face processing and attentional bias (e.g., Gotlib and Joormann, 2010), higher levels of depressive symptoms [Beck Depression Inventory score (BDI) ≥ 17], neurological/psychiatric history, and regular medication use were exclusion criteria; six participants were excluded on this basis, and five more were disqualified from analysis due to extreme error rates (*|z|* > 3; Field, 2009). Analyses included 60 participants (30 women: M_age_ = 20.80, SD = 1.45, range = 18–25 years; M_BDI_ = 6.47, SD = 4.51; 30 men: M_age_ = 21.47, SD = 1.81, range = 19–27 years; M_BDI_ = 8.47, SD = 4.90), all with normal or corrected-to-normal visual acuity. Men and women in Experiment 1 did not differ significantly in either age, *t*(58) = −1.57, *p* = 0.121, or BDI scores, *t*(58) = −1.64, *p* = 0.106.

A sample-size adequacy analysis conducted in G*Power 3.1 (Faul et al., 2007) for a repeated-measures ANOVA interaction effect (within-subject factors; *f* = 0.25, *α* = 0.05) indicated that a minimum sample of 44 participants would provide 90% power to detect a medium-sized interaction effect. The final sample of 60, therefore, exceeded the required sample size.

#### 2.1.2 Materials

##### 2.1.2.1 Beck depression inventory

The BDI (Beck et al., 1961), validated in Turkish by Hisli (1989), is a 21-item self-report measure of depressive symptom severity. Each item consists of four statements reflecting increasing symptom severity, scored from 0 to 3, yielding a total score ranging from 0 to 63; higher scores indicate greater depressive symptom severity. The Turkish version demonstrated adequate internal consistency (Cronbach’s *α* = 0.80; Hisli, 1989).

##### 2.1.2.2 Stimuli

Facial stimuli were selected from the NimStim Set of Facial Expressions (Tottenham et al., 2009), and the same stimulus set was used in both experiments. Previous work has shown that apparent anger- or happiness-superiority effects in visual search can depend substantially on perceptual characteristics of the stimulus set, including the visibility of teeth and whether the mouth is open or closed (Horstmann et al., 2012; Lundqvist et al., 2014; Savage et al., 2016). To reduce this potential salience confound, open-mouth versions were therefore used for both happy and angry expressions. Neutral faces were presented with closed mouths because open-mouth neutral expressions were less reliably recognized, consistent with concerns about perceptual characteristics influencing emotional-face search performance (Pitica et al., 2012).

Stimulus selection was informed by a separate pilot study in which 54 students evaluated 161 photographs of 27 NimStim models for emotion recognition, arousal, and valence. Fourteen models were selected (female models 1, 3, 6, 7, 8, 9, and 10; male models 20, 24, 25, 26, 27, 34, and 36). The final happy and angry expressions showed high recognition accuracy (98.55 and 95.37%, respectively), while neutral expressions were also reliably identified (84.66%). Happy and angry expressions were comparable in rated arousal (M = 6.93 and 7.40, respectively, *p* > 0.05), whereas neutral expressions were lower in arousal (M = 5.06). As intended, happy and angry expressions differed markedly in valence (M = 7.46 and 2.46, respectively, *p* < 0.001), with neutral expressions falling near the midpoint of the scale (M = 4.90). Thus, the selected emotional expressions were comparable in arousal but clearly differentiated in valence.

##### 2.1.2.3 Face discrimination task

A visual-search face discrimination task was programmed in MATLAB R2021b (MathWorks, Natick, MA, USA). The cueing procedure was adapted from Chen and Cave (2013), with their letter-based paradigm modified for emotional face stimuli. Grayscale stimuli were presented against a black background. Each trial began with a 1,000-ms fixation point, followed by a 1,000-ms cue display containing seven oval placeholders: six arranged around an imaginary circle and one at fixation. White placeholders indicated possible target locations, whereas gray placeholders indicated locations at which the target could not appear. In the two-cue condition, two of the six peripheral placeholders were white, and four were gray; in the six-cue condition, all six peripheral placeholders were white. The central placeholder always contained the distractor.

In the stimulus display, two or six peripheral placeholders were replaced by faces, including one happy or angry target and one or more neutral faces. The central distractor was neutral, congruent (the same emotional expression as the target), or incongruent (the opposite emotional expression). Participants categorized the target as happy or angry as quickly and accurately as possible; response mapping to the 1 and 2 keys was counterbalanced across participants (Figure 1A). The stimulus display remained on screen until a response was made. Stimuli and placeholders subtended approximately 3.60° vertically and 3.00° horizontally; peripheral stimuli were positioned 4.49° from fixation at an approximate viewing distance of 60 cm.

**Figure 1:**
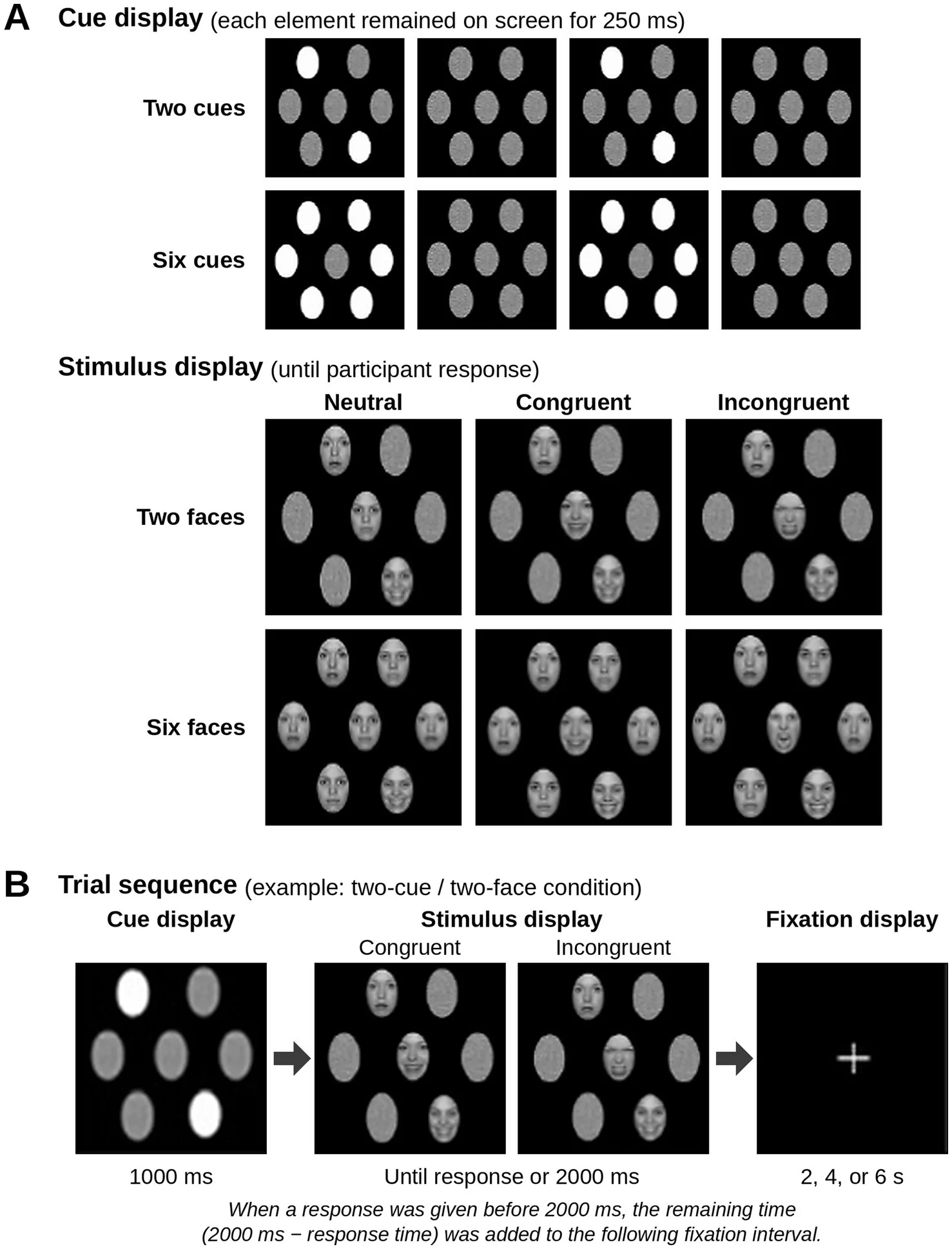
Example stimulus displays and trial procedure. **(A)** Example displays used in Experiment 1, showing the cue display (two/six cues) and the stimulus display (neutral, congruent, and incongruent distractor conditions for two/six faces). **(B)** Example illustration of a single trial in Experiment 2 (fMRI task), showing the cue display, stimulus display (congruent/incongruent), and fixation display, with corresponding trial timing. Face stimuli were taken from the NimStim Set of Facial Expressions (Tottenham et al., 2009). For illustrative purposes, only those models whose images may be reproduced in publications are shown here; consequently, some identities appear more than once within a display. In the actual experiment, every face presented within a display belonged to a different model. Source: NimStim Set of Facial Expressions, Developmental Affective Neuroscience Laboratory, Columbia University.

#### 2.1.3 Procedure

The study was approved by Hacettepe University Ethics Committee (Approval No. 16969557-996), and all participants provided written informed consent. Testing was conducted individually in the Department of Psychology at Hacettepe University and lasted approximately 90 min. After 14 practice trials, participants completed four experimental blocks: two low-load blocks (two possible target locations) and two high-load blocks (six possible target locations), presented in a Latin-square-counterbalanced order. Within each block, face size, target emotion, and distractor type occurred with equal frequency and were presented in randomized order. A total of 672 trials were administered (168 per block), with a 2-min rest interval between blocks. Questionnaires were administered after completion of the experimental task.

#### 2.1.4 Design and analysis

Experiment 1 used a 2 (*cue size*: two, six) × 2 (*face size*: two, six) × 2 (*target emotion*: happy, angry) × 3 (*distractor type*: neutral, congruent, incongruent) repeated-measures design. Cue size operationalized as perceptual load (two cues: low load; six cues: high load), whereas face size operationalized as dilution (two faces: low dilution; six faces: high dilution). To facilitate direct comparison with Experiment 2, the neutral-distractor condition was excluded from the primary congruency analysis. The dependent variable was the reaction time (RT)-based congruency score calculated from correct trials as incongruent RT minus congruent RT. Scores were analyzed using a 2 (cue size) × 2 (face size) × 2 (target emotion) repeated-measures ANOVA with Bonferroni-corrected pairwise comparisons in IBM SPSS Statistics version 23 (IBM Corp., Armonk, NY, USA). Neutral-condition data are reported in Supplementary Table S1.

### 2.2 Experiment 2: fMRI study

#### 2.2.1 Participants

Experiment 2 included 40 female volunteer participants from Hacettepe University who had not participated in Experiment 1. The decision to focus on women was informed by sex-stratified analyses of the Experiment 1 congruency scores, which showed significant cue-related effects in women, including a cue size × target emotion interaction, whereas no significant cue- or face-size effects emerged in men; only a target emotion main effect was significant. This pattern was considered alongside prior evidence of sex differences in emotional expression processing (e.g., Hampson et al., 2006; Stevens and Hamann, 2012), and, in some contexts, greater susceptibility to task-irrelevant information in women (e.g., Judge and Taylor, 2012; Stoet, 2010). Given the limited neuroimaging resources and the primary aim of Experiment 2—to examine the neural correlates of the perceptual load–dilution debate rather than sex differences—the fMRI study therefore focused on a homogeneous female sample. Exclusion criteria were neurological/psychiatric history, relevant medication use, BDI ≥ 17, Handedness Inventory (HI) score >17, MRI-incompatible metal, and refractive error >3 diopters; eight participants were excluded on this basis, and two more were disqualified from analysis due to extreme error rates (|*z*| > 3; Field, 2009). Analyses included 30 healthy right-handed women (M_age_ = 20.73, SD = 1.60, range = 18–25 years; M_BDI_ = 6.73, SD = 3.80), all with normal or corrected-to-normal vision (MRI-compatible glasses where needed). The women in Experiment 2 did not differ significantly from the women in Experiment 1 in either age, *t*(58) = 0.17, *p* = 0.866, or BDI scores, *t*(58) = −0.25, *p* = 0.805.

The target sample size for Experiment 2 was constrained by the substantially greater financial and practical demands of fMRI data collection relative to behavioral testing. A sensitivity analysis in G*Power 3.1 indicated that a sample of 30 participants afforded approximately 76% power to detect a medium-sized within-subject interaction effect (*f* = 0.25, *α* = 0.05). This sample size is within the range of related task-based fMRI studies examining perceptual load and emotional distractor processing (e.g., Bishop et al., 2007; Silvert et al., 2007; Wei et al., 2013), with Wei et al. (2013), for example, using a sample of 16 participants.

#### 2.2.2 Materials

##### 2.2.2.1 Handedness inventory

This 13-item scale (Chapman and Chapman, 1987), adapted into Turkish by Nalçacı et al. (2002), assesses hand preference across everyday activities. Responses are scored to yield a laterality score, with lower scores indicating stronger right-hand preference. To minimize hemispheric-lateralization differences, only right-handed participants (scores ≤ 17) were included.

##### 2.2.2.2 fMRI-adapted face discrimination task

Stimuli and variables matched Experiment 1, except that the neutral-distractor condition was omitted to shorten scan time and isolate the congruency comparison. The task used a mixed design: cue size and face size were manipulated across four blocks (2-cue/2-face, 2-cue/6-face, 6-cue/2-face, and 6-cue/6-face) to maintain a consistent attentional set within each cue-size × face-size condition (e.g., Chen and Cave, 2013), whereas target emotion and distractor type were randomized within sub-blocks. Each trial began with a jittered fixation interval of 2, 4, or 6 s (60, 30, and 10% of trials, respectively), followed by a 1,000-ms cue display and a stimulus display lasting up to 2,000 ms. If a response occurred before 2,000 ms, fixation was presented for the remainder of the interval to maintain a constant 2,000-ms post-cue response interval (Figure 1B).

#### 2.2.3 fMRI data acquisition

Scans were acquired at the National Magnetic Resonance Research Center (UMRAM) on a 3T Siemens Magnetom Trio with a 32-channel head coil. T1-weighted anatomical images came first (TR = 2,600 ms, TE = 2.92 ms, FA = 12°, FOV = 256 mm, matrix = 256 × 256, slice thickness = 1 mm, voxel size = 1 × 1 × 1 mm), followed by T2*-weighted EPI functional images (TR = 2000 ms, TE = 30 ms, FA = 90°, GRAPPA = 2, FOV = 192 mm, matrix = 64 × 64, 35 slices, slice thickness = 3 mm, voxel size = 3 × 3 × 3 mm). The structural scan lasted approximately 6 min. Functional data were acquired in two or three 11-min runs, as described below. Image preprocessing and statistical analyses were performed in SPM12 (Wellcome Trust Centre for Neuroimaging, 2014).

#### 2.2.4 Procedure

Ethics approval and consent procedures were the same as in Experiment 1. Questionnaires were administered in the Department of Psychology at Hacettepe University; the fMRI task was administered at UMRAM. During screening, the BDI and HI were administered, and eligible participants proceeded to the scanning phase.

Participants completed 24 practice trials before scanning. Following the structural scan, participants completed two (*n* = 17) or three (*n* = 13) 11-min functional runs, depending on fatigue. Each run contained 96 trials organized into the four cue-size × face-size blocks, with three 8-trial sub-blocks per block. Block order was counterbalanced using a Latin-square arrangement. A 3-s reminder of the response mapping was presented between sub-blocks, and a 20-s fixation interval separated the main blocks. Each run began and ended with a 6-s fixation period. Thus, participants completed 192 or 288 trials, depending on the number of functional runs. Response mapping to the blue and yellow buttons was counterbalanced across participants (Figure 2).

**Figure 2:**
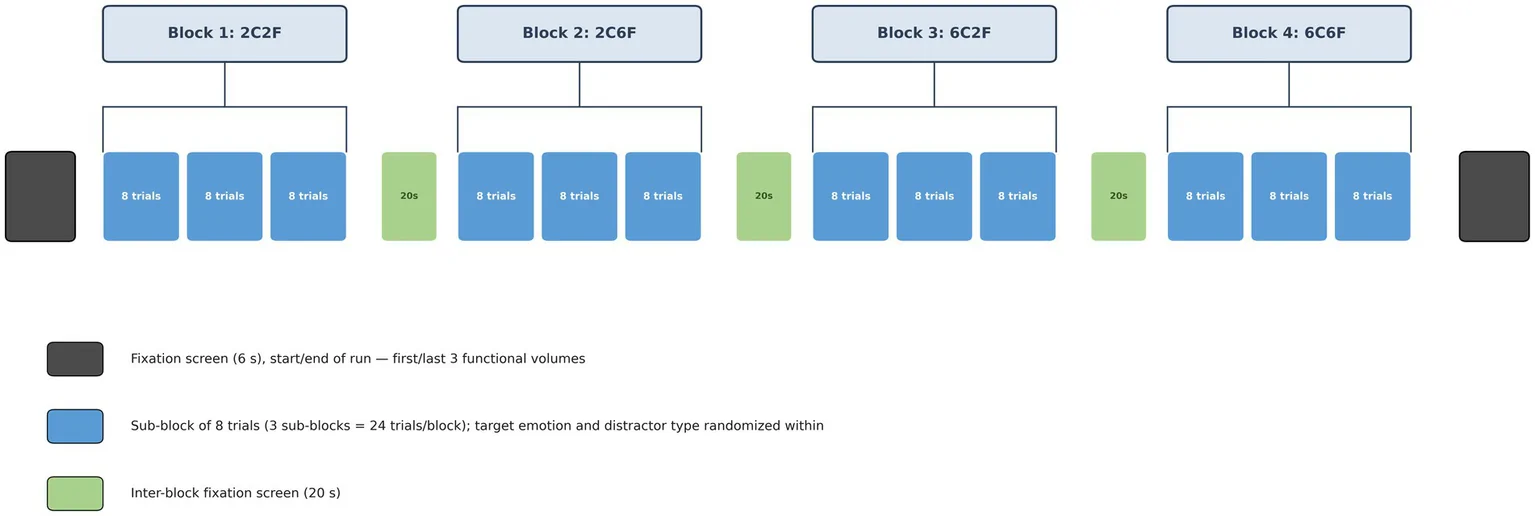
Schematic illustration of the fMRI task block structure (Experiment 2). Each of the four blocks (2C2F, 2C6F, 6C2F, 6C6F) comprised three sub-blocks of eight trials (24 trials/block), separated by 20-s fixation periods, with 6-s fixation periods at the beginning and end of the run.

#### 2.2.5 Design and analysis

Experiment 2 used a 2 (*cue size:* two, six) × 2 (*face size*: two, six) × 2 (*target emotion*: happy, angry) × 2 (*distractor type*: congruent, incongruent) repeated-measures design, with RT-based congruency scores and BOLD signal change as dependent variables. Behavioral data were analyzed as in Experiment 1. Functional images were preprocessed in SPM12 using realignment, co-registration, slice-timing correction, segmentation, MNI normalization, and 12-mm FWHM smoothing.

Each of the 16 experimental conditions was modeled as a separate event-related regressor (duration = 0 s), time-locked to stimulus onset. First-level models used the canonical hemodynamic response function, a 128-s high-pass filter, and an AR(1) model to account for serial correlations. Six head-motion parameters were included as nuisance regressors in each functional session, with each run modeled as a separate session and its own session-specific constant.

Second-level analyses were performed in SPM12 using a full factorial model comprising the 16 cells defined by cue size, face size, target emotion, and distractor type (2 × 2 × 2 × 2), with all 30 participants contributing observations to each cell. Whole-brain omnibus *F*-tests were conducted for all main effects and interactions. Primary directional *T*-contrasts characterized the direction of each significant omnibus main effect (six > two cues, six > two faces, angry > happy, and incongruent > congruent); consistent with the non-directional prediction stated in the Introduction, the angry > happy direction was selected *post hoc* to characterize the significant omnibus emotion effect rather than to test an *a priori* valence-based hypothesis, and the reverse-direction contrast is reported in Supplementary Table S2. Significant interactions were characterized using condition-specific simple-effect contrasts; these follow-ups were used to describe the source of omnibus effects rather than as independent tests of differences between thresholded maps. Complete directional and simple-effect results are reported in the Supplementary Table S2. Monte Carlo cluster-extent correction (10,000 iterations; Slotnick, 2017) used a voxelwise threshold of *p* < 0.001 and cluster extent of *k* ≥ 18 (corrected cluster-level *α* = 0.05). Whole-brain statistical maps were visualized using nilearn (Abraham et al., 2014; Nilearn contributors, 2023). Anatomical region labels were assigned using the SPM12 version of the Automated Anatomical Labeling atlas (AAL; Tzourio-Mazoyer et al., 2002), whereas Brodmann areas were identified using the Brodmann-area level of the Talairach atlas (Lancaster et al., 2000), both accessed through nilearn (Abraham et al., 2014).

To assess the potential influence of unequal scanning exposure, additional robustness analyses were conducted. First, the Experiment 2 behavioral repeated-measures ANOVA was repeated as a repeated-measures ANCOVA with the number of completed functional runs entered as a covariate. Second, the whole-brain second-level model was repeated with run count included as a covariate. Finally, an equal-exposure sensitivity analysis was performed by re-estimating first-level models using only the first two functional runs available for all participants and repeating the second-level whole-brain analyses. Robustness results are reported in Supplementary Tables S3–S5.

Region-of-interest (ROI) analyses included bilateral AMG, ACC, DLPFC, and FEF. Each ROI was defined in MarsBaR (Brett et al., 2002) as a 10-mm-radius sphere centered on an MNI coordinate derived by averaging previously reported peak coordinates. The resulting centers were ACC: right 4, 20, 40; left −4, 20, 40 (Mitchell, 2005; Wei et al., 2013); DLPFC: right 43, 27, 26; left −42, 34, 23 (Mitchell, 2005; Shafer et al., 2012); FEF: right 26, −4, 54; left −27, −7, 54 (Donner et al., 2000; Heinen et al., 2014); AMG: right 27, −3, −19; left −25, −5, −19 (Kätsyri et al., 2020; Peluso et al., 2009; Silvert et al., 2007). Mean percent signal change (PSC) was extracted from each ROI and entered into repeated-measures ANOVAs. Significant interactions were decomposed with Bonferroni-corrected pairwise comparisons. PSC extraction was performed in Python (Python Software Foundation, Wilmington, DE, USA), and ROI analyses were performed in IBM SPSS Statistics version 23.

## 3 Results

### 3.1 Experiment 1

Because error rates were very low, only RT findings are reported, computed from correctly identified targets and excluding responses faster than 200 ms or slower than 2000 ms, consistent with standard practice in visual search paradigms (1.19% of all data) (e.g., Biggs and Gibson, 2014; Chen and Cave, 2013). For comparability with Experiment 2 and the literature, distractor interference was assessed using the congruency score (incongruent-RT − congruent-RT), consistent with approaches that quantify interference magnitude as a composite score. A 2 (cue size) × 2 (face size) × 2 (target emotion) repeated-measures ANOVA with Bonferroni-corrected pairwise comparisons was used. Raw RT means for all conditions, including neutral, appear in Supplementary Table S1.

The congruency score showed significant main effects of cue size [*F*(1, 59) = 7.29, *p* = 0.009, *η^2^_p_* = 0.11] and target emotion [*F*(1, 59) = 11.55, *p* = 0.001, *η^2^_p_* = 0.16]. The congruency effect was larger for six cues (26.76 ± 4.68 ms) than for two (11.55 ± 3.12 ms), indicating a reversed load effect—interference susceptibility increased with load. It was also larger for happy targets (28.47 ± 3.78 ms) than for angry targets (9.84 ± 4.06 ms): congruency-related interference was greater for happy than for angry targets.

A significant cue size × target emotion interaction [*F*(1, 59) = 7.56, *p* = 0.008, *η^2^_p_* = 0.11] indicated target emotion’s effect depended on load: happy (12.74 ± 4.21 ms) and angry (10.36 ± 4.53 ms) congruency scores did not differ at two cues (diff = 2.39 ms, *p* = 0.699) but did at six cues (happy: 44.20 ± 6.83 ms; angry: 9.32 ± 6.58 ms; diff = 34.88 ms, *p* = 0.001). Importantly, for happy targets, congruency scores were significantly greater in the six-cue than in the two-cue condition (diff = 31.83 ms, Bonferroni-adjusted *p* < 0.001), whereas cue size had no significant effect for angry targets (diff = 0.94 ms, *p* = 0.905; Figure 3). Thus, the reversed cue-size effect on distractor interference was specific to happy targets.

**Figure 3:**
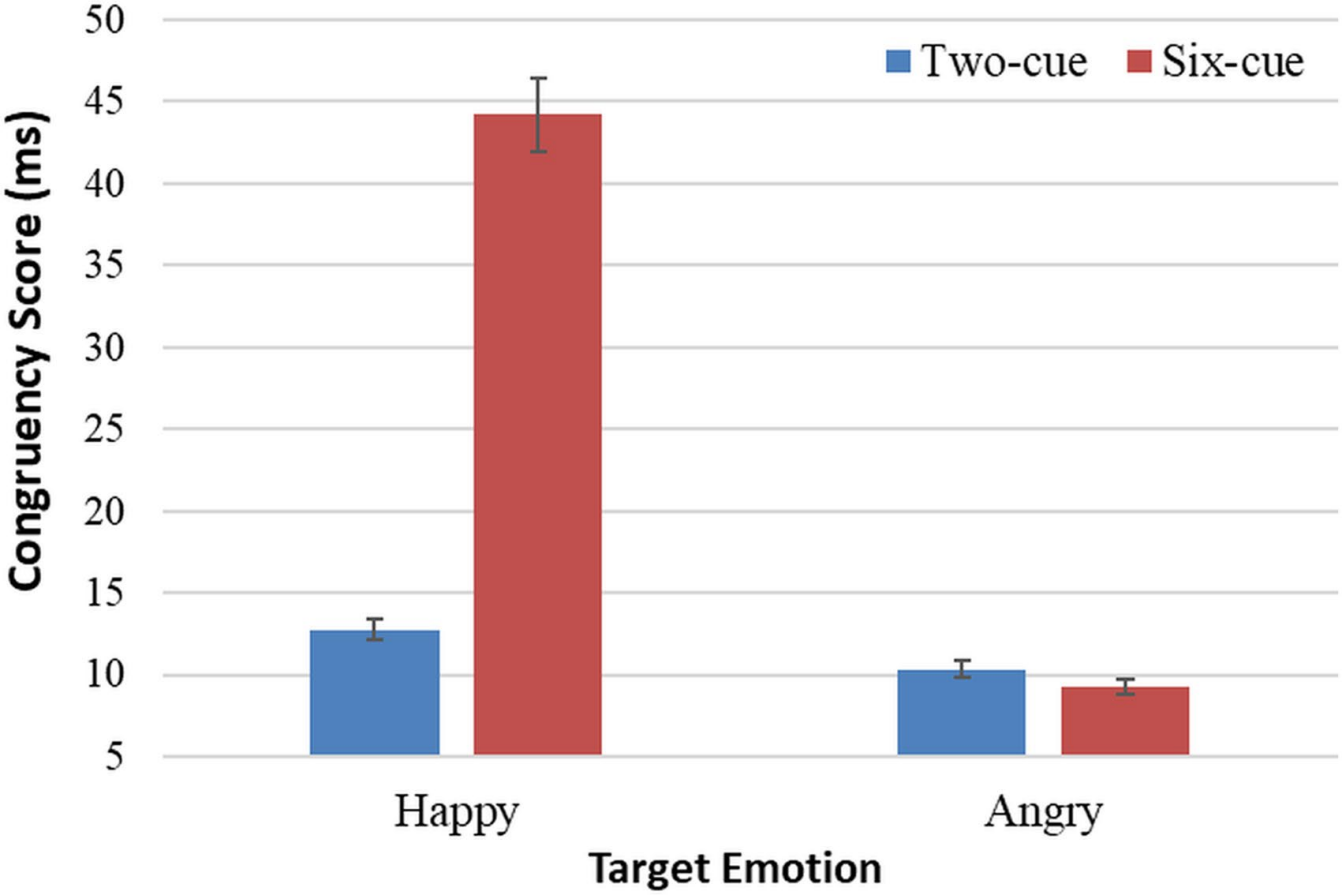
Congruency effects as a function of target emotion and cue size in Experiment 1. Higher values indicate greater interference from incongruent relative to congruent distractors. Error bars represent SEM.

Face size showed no significant main or interaction effects (*p* > 0.05); no dilution effect emerged. In summary, distractor interference rose with PL and only for happy targets, but was unaffected by the number of neutral faces on screen (dilution).

### 3.2 Experiment 2

#### 3.2.1 Behavioral findings

As in Experiment 1, only RT-based analyses are reported (1.02% excluded), using identical criteria and correction procedures. Interestingly, no main or interaction effects on the congruency score were significant (all *p*_s_ > 0.05), and no significant modulation of congruency scores by cue size, face size, or target emotion was detected under MRI conditions. Additionally, to assess whether behavioral performance varied as a function of scanning exposure, this analysis was repeated as a repeated-measures ANCOVA with the number of completed functional runs included as a covariate. Run count was not significantly associated with overall behavioral performance, *F*(1, 28) = 0.06, *p* = 0.813, *η^2^_p_* = 0.002, and none of the interactions involving run count reached significance (all *p*_s_ ≥ 0.05; see Supplementary Table S5).

#### 3.2.2 Neuroimaging findings

Whole-brain omnibus *F*-tests evaluated all main effects and interactions. Table 1 reports all effects that reached significance. Among the corresponding follow-up *T*-contrasts, Table 2 reports the four main-effect directional contrasts together with all condition-specific congruency contrasts (incongruent > congruent) derived from significant interactions involving distractor type. The complete set of directional and condition-specific *T*-contrasts is provided in Supplementary Table S2.

**Table 1:** Whole-brain omnibus *F*-test results (Experiment 2).

Effect	Region	Hemi	BA	*x*	*y*	*z*	*k*	*F*
Cue size	Calcarine sulcus	R	BA17	20	−72	12	1,324	24.57
	Superior frontal gyrus, dorsolateral	R	BA6	28	−10	70	32	16.08
Face size	Paracentral lobule	L	BA5	−2	−32	56	19	14.32
	Precentral gyrus	L	BA6	−38	−8	58	94	14.93
Target emotion	Cerebellum	L	–	−28	−70	−18	10,258	40.96
	Frontal superior orbital	R	BA47	18	30	−10	18	19.11
	Inferior parietal gyrus	L	BA7	−28	−50	40	407	17.61
	Superior frontal gyrus, medial	L	BA6	−6	30	36	111	17.31
	Superior occipital gyrus	R	BA7	28	−66	42	142	14.92
	Middle frontal gyrus	R	BA6	38	4	52	36	13.36
Distractor type	Calcarine sulcus	R	BA17	20	−92	−4	8,820	51.80
	Precuneus	R	BA7	6	−58	64	175	16.72
	Superior parietal gyrus	L	BA7	−24	−52	50	131	16.63
	Superior occipital gyrus	R	BA39	28	−60	36	79	15.34
Face × emotion	Thalamus (pulvinar)	R	–	4	−26	10	27	16.27
	Rolandic operculum	R	BA43	54	−16	20	40	14.29
Face × type	Precuneus	R	BA7	18	−58	44	49	13.72
Emotion × type	Precentral gyrus	R	BA4	42	−14	56	132	18.95
	Lingual gyrus	R	BA30	8	−60	8	200	15.49
Face × emotion × type	Middle temporal gyrus	R	BA21	62	−8	−14	24	13.97
Cue × face × type	Paracentral lobule	R	BA6	12	−31	58	26	18.44
	Superior temporal gyrus	R	BA22	62	−50	20	178	17.79
	Putamen	R	–	32	8	10	44	17.51
	Supramarginal gyrus	L	BA40	−64	−28	28	55	15.20
	Precentral gyrus	R	BA6	50	−2	26	42	13.27

Peak MNI coordinates and cluster statistics for the omnibus effects emphasized in the main text. All clusters survived voxelwise *p* < 0.001 (uncorrected) and cluster extent *k* ≥ 18 voxels (Monte Carlo correction). BA, Brodmann area (−, subcortical/non-cortical); Hemi, hemisphere; *k*, cluster size in voxels; *F*, peak *F*-value.

**Table 2:** Whole-brain *T*-contrast results (Experiment 2).

Contrast	Region	Hemi	BA	*x*	*y*	*z*	*k*	*T*
6-cue > 2-cue	Calcarine sulcus	R	BA17	20	−72	12	1745	4.96
	Superior frontal gyrus, dorsolateral	R	BA6	28	−10	70	47	3.97
	Inferior parietal gyrus	R	BA40	38	−54	42	42	3.64
	Cerebellum, crus I	L	–	−18	−74	−30	24	3.54
	Inferior frontal gyrus, opercular part	R	BA9	38	8	32	41	3.50
6-face > 2-face	Paracentral lobule	L	BA5	−2	−32	56	32	3.78
Angry > happy	Superior frontal gyrus, medial	L	BA6	−6	30	36	170	4.16
	Frontal mid orbital	L	BA10	−36	48	−4	29	3.58
Incongruent > congruent	Calcarine sulcus	R	BA17	20	−92	−2	10,155	7.20
	Hippocampus	L	BA27	−20	−30	−4	22	4.31
	Superior parietal gyrus	L	BA7	−24	−52	50	255	4.08
	Putamen	L	–	−24	4	8	26	3.72
	Precentral gyrus	R	BA6	44	0	24	31	3.68
2-cue 2-face: incongruent > congruent	Rolandic operculum	R	BA13	40	−4	16	119	4.25
2-cue 6-face: incongruent > congruent	Lingual gyrus	R	BA18	20	−88	−12	4,989	5.32
	Superior occipital gyrus	R	BA7	26	−62	44	389	4.38
	Globus pallidus	L	–	−20	0	6	107	4.15
	Superior parietal gyrus	L	BA7	−22	−58	48	192	4.11
	Superior occipital gyrus	R	BA19	22	−84	36	61	3.81
	Middle frontal gyrus	R	BA6	34	−2	52	66	3.63
6-cue 2-face: incongruent > congruent	Lingual gyrus	L	BA18	−10	−74	−2	2,198	4.92
	Thalamus	L	–	−4	−26	−8	69	3.73
	Lingual gyrus	R	BA18	24	−90	−6	110	3.63
	Fusiform gyrus	R	–	30	−64	−16	23	3.55
6-cue 6-face: incongruent > congruent	Calcarine sulcus	R	BA17	22	−94	2	706	4.71
	Inferior temporal gyrus	L	BA37	−48	−46	−14	149	4.34
	Fusiform gyrus	L	BA19	−34	−70	−14	169	4.13
	Angular gyrus	R	–	30	−48	34	55	3.94
2-face: incongruent > congruent	Lingual gyrus	R	BA18	24	−90	−4	2,468	4.41
	Fusiform gyrus	R	BA19	28	−64	−14	100	3.71
6-face: incongruent > congruent	Calcarine sulcus	R	BA17	16	−94	−2	7,829	6.17
	Superior parietal gyrus	L	BA7	−22	−54	48	366	4.62
	Hippocampus	L	BA27	−22	−28	−4	23	4.07
Angry: incongruent > congruent	Lingual gyrus	L	BA18	−10	−88	−12	1,558	4.57
	Fusiform gyrus	R	BA20	42	−34	−18	38	3.95
	Fusiform gyrus	R	BA19	30	−60	−12	74	3.65
Happy: incongruent > congruent	Lingual gyrus	R	BA18	16	−90	−12	8,866	6.61
	Globus pallidus	L	–	−20	−4	4	98	4.40
	Middle frontal gyrus	R	BA6	40	−8	54	191	4.24
	Precentral gyrus	L	BA4	−38	−14	48	231	3.94
	Superior parietal gyrus	L	BA7	−22	−68	42	207	3.84
2-face angry: incongruent > congruent	Calcarine sulcus	R	BA17	12	−64	8	1,198	4.33
	Precentral gyrus	L	BA4	−36	−12	52	152	3.79
	Superior temporal gyrus	R	BA22	70	−22	0	45	3.78
	Cerebellum, crus I	L	–	−20	−80	−20	126	3.63
	Lingual gyrus	R	BA18	14	−88	−14	49	3.58
	Precentral gyrus	R	BA4	40	−12	50	37	3.57
2-face happy: incongruent > congruent	Superior frontal gyrus, medial	L	BA6	−8	28	36	54	3.96
	Inferior frontal gyrus, triangular part	L	BA10	−38	40	0	124	3.94
	Fusiform gyrus	R	BA19	30	−60	−8	56	3.72
	Frontal superior orbital	L	BA10	−34	60	−2	20	3.62
	Lingual gyrus	R	BA18	22	−90	−6	35	3.49
6-face angry: incongruent > congruent	Lingual gyrus	R	BA18	22	−88	−10	8,272	6.02
	Superior parietal gyrus	L	BA7	−22	−56	46	409	4.25
	Middle frontal gyrus	R	BA6	36	0	54	93	3.74
6-face happy: incongruent > congruent	Calcarine sulcus	L	BA18	−10	−90	−10	328	3.93
	Thalamus (pulvinar)	L	–	−10	−24	6	25	3.83

Peak MNI coordinates and cluster statistics for the four main-effect directional contrasts and all condition-specific congruency contrasts (incongruent > congruent) derived from significant omnibus interactions involving distractor type. *T*-contrasts for interactions not involving distractor type, and the reverse-direction contrasts, were not systematically tested. The complete set of directional *T*-contrasts is reported in Supplementary Table S2. All clusters survived voxelwise *p* < 0.001 (uncorrected) and cluster extent *k* ≥ 18 voxels (Monte Carlo correction). BA, Brodmann area; Hemi, hemisphere; *k*, cluster size in voxels; *T*, peak *T*-value.

*ROI-related percent signal change (PSC) results* (Tables 3, 4). Congruency was significant across all eight *a priori* ROIs, with higher PSC for incongruent than for congruent trials in bilateral ACC, AMG, DLPFC, and FEF (*ηp*^2^ = 0.166–0.315). Cue size significantly affected only the left FEF, where PSC was greater for two than for six cues. Higher-order effects were more selective. cue size × face size × target emotion interactions emerged in the left ACC and right AMG, whereas the left DLPFC showed both a target emotion × congruency interaction and a cue size × target emotion × congruency interaction. Bonferroni-corrected follow-up comparisons showed happy > angry responses in the two-cue/six-face condition in the left ACC and right AMG. In the left DLPFC, incongruent > congruent differences were significant for happy targets overall, for happy targets under two cues, and for angry targets under six cues (Table 4).

**Table 3:** Region-of-interest (ROI) omnibus *F*-test results (Experiment 2).

Effect	ROI	*F*	*p*	*η* ^2^ *_p_*
Congruency	ACC_L	9.57	0.004	0.248
	ACC_R	12.34	0.001	0.299
	AMG_L	7.37	0.011	0.203
	AMG_R	5.77	0.023	0.166
	DLPFC_L	9.39	0.005	0.245
	DLPFC_R	13.31	0.001	0.315
	FEF_L	10.26	0.003	0.261
	FEF_R	8.87	0.006	0.234
Cue size	FEF_L	5.12	0.031	0.150
Cue × face × emotion	ACC_L	4.31	0.047	0.129
	AMG_R	4.52	0.042	0.135
Emotion × congruency	DLPFC_L	4.33	0.046	0.130
Cue × emotion × congruency	DLPFC_L	5.24	0.030	0.153

Repeated-measures ANOVA results for percent signal change; only effects reaching significance (*p* < 0.05) are shown. df = 1, 29 for all tests. ACC, anterior cingulate cortex; AMG, amygdala; DLPFC, dorsolateral prefrontal cortex; FEF, frontal eye fields; L, left; R, right; *η*^2^*_p_*, partial eta squared.

**Table 4:** ROI follow-up comparisons for significant omnibus effects (Experiment 2).

Contrast	ROI	Mean diff.	95% CI	*t*	*p*
Incongruent > congruent	ACC_L	0.020	(0.007, 0.033)	3.09	0.004
	ACC_R	0.025	(0.010, 0.039)	3.51	0.001
	AMG_L	0.022	(0.005, 0.039)	2.71	0.011
	AMG_R	0.017	(0.002, 0.031)	2.40	0.023
	DLPFC_L	0.027	(0.009, 0.046)	3.06	0.005
	DLPFC_R	0.031	(0.014, 0.048)	3.65	0.001
	FEF_L	0.023	(0.008, 0.038)	3.20	0.003
	FEF_R	0.019	(0.006, 0.033)	2.98	0.006
2-cue > 6-cue	FEF_L	0.026	(0.003, 0.050)	2.26	0.031
2-cue 6-face: happy > angry	ACC_L	0.036	(0.010, 0.062)	2.87	0.008
	AMG_R	0.031	(0.003, 0.058)	2.30	0.029
Happy: incongruent > congruent	DLPFC_L	0.039	(0.019, 0.060)	3.90	<0.001
2-cue happy: incongruent > congruent	DLPFC_L	0.052	(0.023, 0.082)	3.61	0.001
6-cue angry: incongruent > congruent	DLPFC_L	0.035	(0.006, 0.065)	2.45	0.021

Follow-up pairwise comparisons were Bonferroni-corrected; only significant comparisons are shown. df = 29 for all tests. Mean differences and 95% CIs are expressed in percent signal change. The cue size comparison is reported in the observed direction (2-cue > 6-cue).

Whole-brain results (Tables 1, 2; Figure 4). Cue size significantly modulated activity in the right calcarine sulcus and right dorsolateral superior frontal gyrus. In the six-cue > two-cue direction, additional clusters were observed in the right inferior parietal gyrus, right inferior frontal gyrus, and left cerebellar Crus I (Figure 4A). Face size produced a more restricted pattern, with significant omnibus effects in the left paracentral lobule and precentral gyrus; the six-face > two-face contrast yielded a significant cluster in the left paracentral lobule (Figure 4B). Target emotion produced significant effects across cerebellar, frontal, parietal, and occipital regions, whereas the angry > happy contrast was associated primarily with medial and orbital frontal activity (Figure 4C). Distractor type produced the most extensive whole-brain effect, including visual, parietal, and precuneus regions. The incongruent > congruent contrast additionally revealed significant activity in the right calcarine sulcus, left hippocampus, left superior parietal gyrus, left putamen, and right precentral gyrus (Figure 4D).

**Figure 4:**
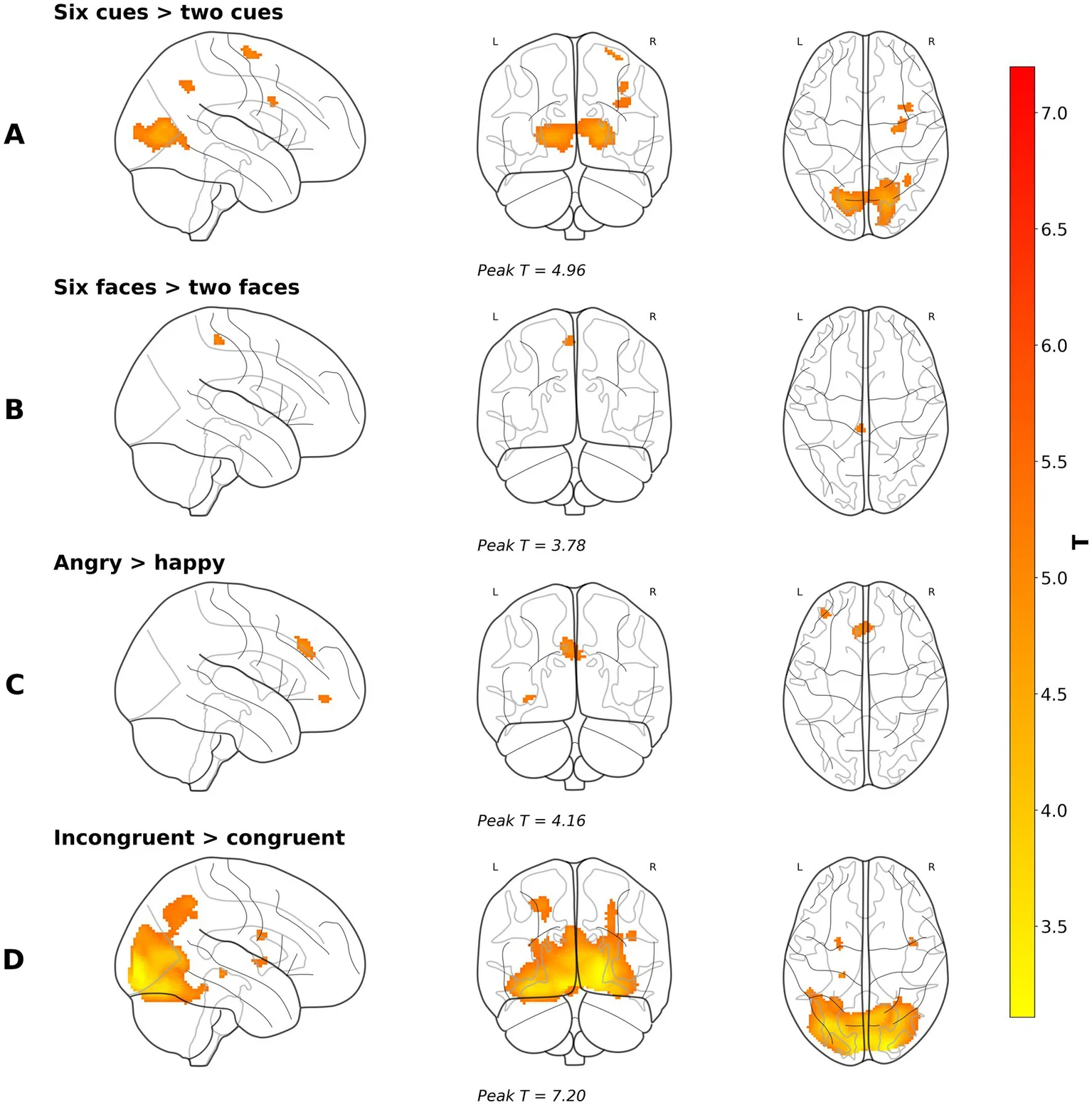
Glass-brain *T*-statistic maps for the four main-effect contrasts. **(A)** Six cues > two cues. **(B)** Six faces > two faces. **(C)** Angry > happy. **(D)** Incongruent > congruent. Maps are thresholded at voxelwise *p* < 0.001 uncorrected (*T* > 3.108) with a cluster-extent threshold of *k* ≥ 18 voxels (Monte Carlo simulation, Slotnick method), consistent with the criteria applied throughout this study; only the hypothesized directional effect is shown. The color bar reflects a shared *T*-value scale across panels; peak *T*-values are reported below each panel. L, left; R, right.

Significant whole-brain interactions further indicated that the neural expression of distractor incongruency depended on the broader task configuration. A cue size × face size × distractor Type interaction involved right paracentral and precentral regions, right superior temporal gyrus and putamen, and left supramarginal gyrus. Condition-specific incongruent > congruent contrasts showed different spatial distributions across the four cue-size × face-size combinations (Figure 5). A target emotion × distractor type interaction was observed in the right precentral and lingual gyri; corresponding simple-effect maps showed significant incongruency-related activation for both angry and happy targets (Figure 6). Additional significant interactions included face size × target emotion in the right thalamus/pulvinar and rolandic operculum, face size × distractor type in the right precuneus, and face size × target emotion × distractor type in the right middle temporal gyrus. These condition-specific maps characterize the significant omnibus interactions and should not be interpreted as direct statistical comparisons between separately thresholded maps. Robustness analyses generally supported the primary whole-brain findings. Including run count as a covariate preserved the overall pattern of omnibus effects, including the critical cue size × face size × distractor type interaction. In the equal-exposure analysis restricted to the first two functional runs, seven of the nine omnibus effects were retained, including the cue size × face size × distractor type interaction, whereas the face main effect and the face size × target emotion × distractor type interaction were not retained (for details, see Supplementary Tables S3–S4).

**Figure 5:**
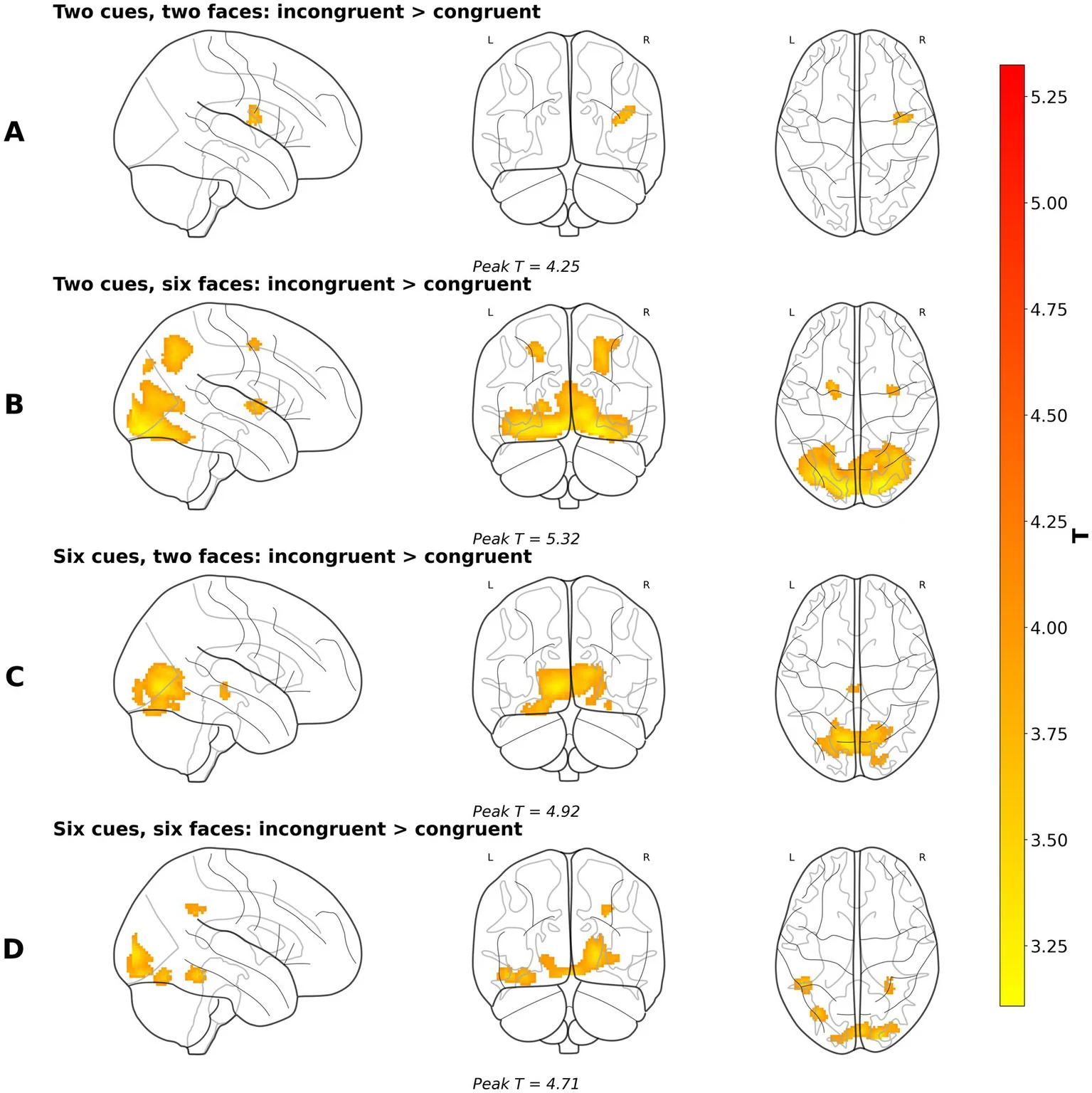
Glass-brain *T*-statistic maps for the congruency effect (incongruent > congruent), decomposed by cue-size × face-size combination. **(A)** Two cues, two faces. **(B)** Two cues, six faces. **(C)** Six cues, two faces. **(D)** Six cues, six faces. These simple effects follow up the significant cue × face × distractor-type interaction (see Table 1). Maps are thresholded at voxelwise *p* < 0.001 uncorrected (*T* > 3.108) with a cluster-extent threshold of *k* ≥ 18 voxels; color bar reflects a shared *T*-value scale across panels, with peak *T*-values reported below each panel. L, left; R, right.

**Figure 6:**
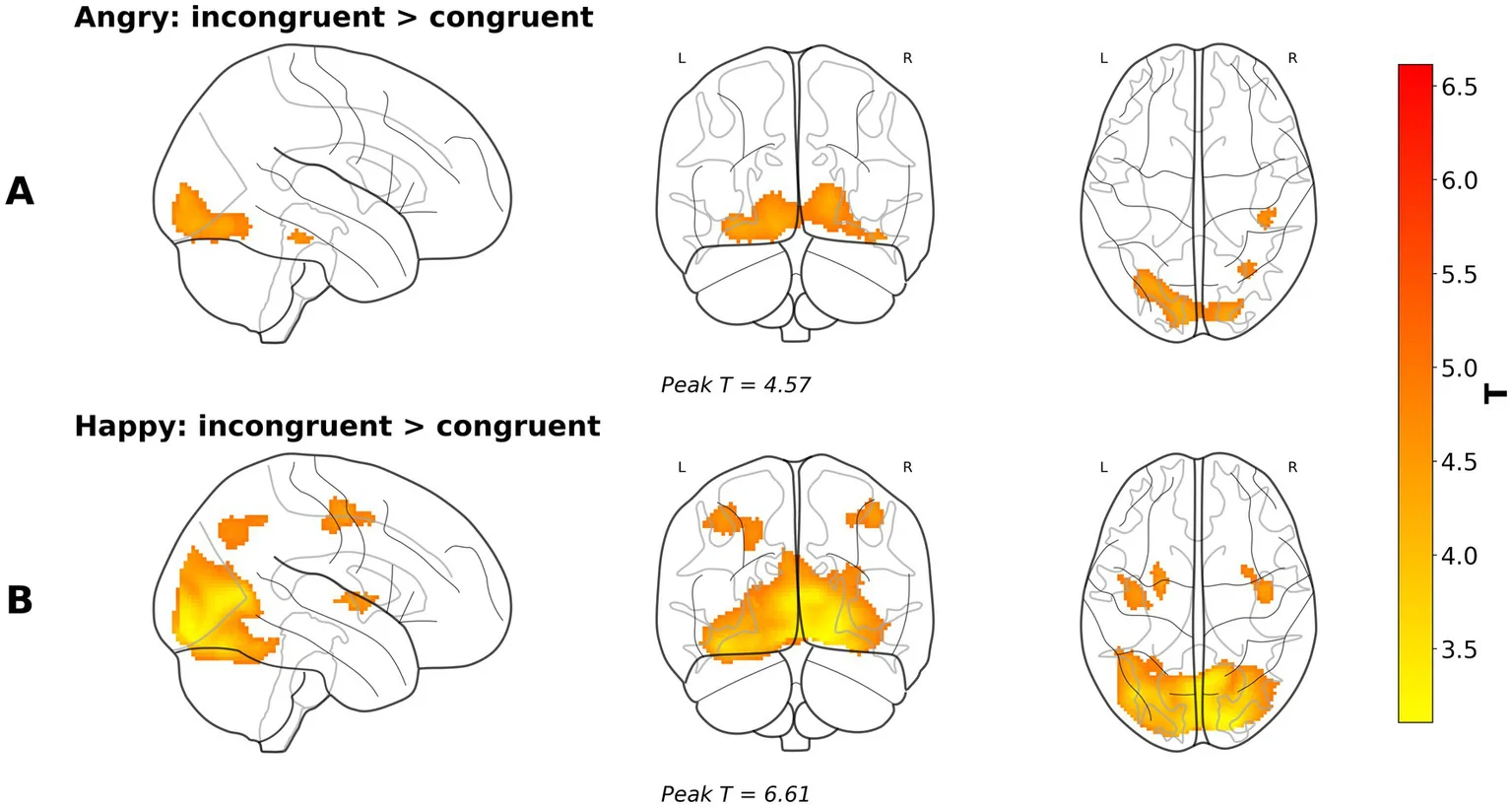
Glass-brain *T*-statistic maps for the congruency effect (incongruent > congruent), decomposed by target emotion. **(A)** Angry targets. **(B)** Happy targets. These simple effects follow up the significant Emotion × Distractor-type interaction (see Table 1). Maps are thresholded at voxelwise *p* < 0.001 uncorrected (*T* > 3.108) with a cluster-extent threshold of *k* ≥ 18 voxels; color bar reflects a shared *T*-value scale across panels, with peak *T*-values reported below each panel. L, left; R, right.

Taken together, the whole-brain and ROI analyses showed robust neural sensitivity to distractor incongruency, whereas cue size, face size, and target emotion produced more selective and context-dependent modulation.

## 4 Discussion

### 4.1 Behavioral evidence for cue-based demand and display dilution

This study tested the PL–dilution debate with emotional faces while minimizing the target–distractor category confound that has complicated prior work. Three findings refine this debate in the context of emotional distraction. First, increasing cue-based target uncertainty enhanced rather than reduced behavioral distractor interference, selectively for happy targets. Second, increasing the number of neutral faces produced no corresponding reduction in interference, despite longer overall response times. Third, cue size and face size yielded non-equivalent neural profiles, while neural sensitivity to distractor incongruency remained robust across affective and attentional-control systems even when behavioral modulation was weak in the scanner. Together, these findings argue against treating cue-based attentional demand and display composition as interchangeable manipulations and instead point to a context-dependent interaction among spatial uncertainty, representational competition, and emotional relevance.

The increase in interference with cue size is inconsistent with the central prediction of classical Load Theory, which anticipates less distractor processing as task-relevant perceptual demands increase (Lavie, 1995, 2005; Lavie and Tsal, 1994). Instead, it resembles the reversed load effect reported when load and dilution are separated (Benoni and Tsal, 2010; Tsal and Benoni, 2010; Benoni, 2025). Face size, by contrast, did not influence interference. This pattern is broadly consistent with Chen and Cave’s (2013) Experiment 3, in which neutral items sharing low-level visual features but not the target–distractor representational structure failed to produce dilution. They proposed that when task performance depends on categorical or semantic processing, dilution may arise beyond the feature level. Likewise, the neutral fillers in the present task differed from the emotional target–distractor pair in expression, emotional meaning, and mouth configuration, and therefore may not have competed at the representational level needed to weaken distractor processing. This interpretation is also compatible with evidence that perceptual grouping and the representational relationship among display items shape dilution (Benoni and Tsal, 2010; Yeh and Lin, 2013). Importantly, the behavioral null for face size does not imply neural irrelevance: Face size entered higher-order interactions involving target emotion and distractor congruency.

The emotion dependence of the reversed load effect further constrains a simple capacity account. Congruency scores for angry targets remained relatively low across cue conditions, whereas interference increased markedly for happy targets under six cues. This asymmetry should not be interpreted as a generalized superiority of angry expressions. Rather, angry targets appeared relatively resistant to the cue-related increase in interference that affected happy targets, while angry distractors were more disruptive when the target was happy. Such a pattern is compatible with face-in-the-crowd accounts of threat prioritization (Öhman et al., 2001) but also with evidence that apparent advantages for anger and happiness depend strongly on the stimulus set and task demands (Savage et al., 2016). In the present paradigm, target emotion therefore altered susceptibility to distraction rather than producing a uniform valence advantage.

More broadly, these findings support the view that perceptual load is not a unitary determinant of distractor processing. Chen and Cave (2013) emphasized that target-location uncertainty, attentional focus, and stimulus category can each independently alter distractor interference, regardless of nominal load. The present findings extend this argument to emotional stimuli: increasing cue-based uncertainty enhanced rather than suppressed interference for happy targets, whereas increasing the number of neutral faces produced no corresponding dilution effect. Thus, the consequences of increasing visual demand appear to depend on how that demand is instantiated and on the representational relationship among targets, fillers, and distractors, rather than on perceptual load alone.

### 4.2 Neural consequences of cue size and face size

Cue size and face size were also associated with distinct neural profiles, consistent with the behavioral dissociation observed in Experiment 1, although the neural and behavioral effects were established in different experiments and should not be equated. Cue size affected the right calcarine cortex and a dorsal superior frontal region, and the six-cue > two-cue contrast additionally involved inferior parietal and inferior frontal regions. This profile is broadly consistent with increased visual selection and spatial uncertainty when several potential target locations must be monitored (Wei et al., 2013; Mitchell, 2005; Donner et al., 2000; Heinen et al., 2014). In the left FEF ROI, however, PSC was higher for two than six cues. Given the role of the FEF in spatial selection, this may reflect more focused, task-relevant selection when potential target locations were restricted, although this interpretation remains tentative (Donner et al., 2000; Heinen et al., 2014). In the primary analysis, face size was associated with relatively restricted paracentral and precentral effects and showed no significant main effect in the *a priori* ROIs. However, the whole-brain face-size main effect was not retained in the equal-exposure two-run sensitivity analysis. Thus, the apparent neural asymmetry between cue size and face size should be interpreted with caution; nevertheless, the weaker face-size effects, together with the more consistent cue-size effects, still suggest that the two manipulations were not neurally interchangeable. Here, however, cue-based uncertainty altered both behavioral interference and a broader visual–attentional profile, whereas increasing display density did neither in a comparable manner. Thus, the present findings suggest that the behavioral distinction between load and dilution is accompanied by a corresponding difference in their neural expression. This pattern extends the distinction emphasized by Wei et al. (2013): increasing attentional demand and increasing the number of display items need not produce equivalent effects.

The present pattern may also be compatible with computational accounts in which perceptual load and dilution emerge from interacting competitive processes rather than from wholly separate mechanisms. Neokleous et al. (2016), for example, modeled distractor processing as the joint outcome of stimulus-driven competition and top-down attentional biasing. From this perspective, the non-equivalent yet interacting effects of cue-based uncertainty and display composition observed here may reflect changes in the balance among competing representations rather than two entirely independent mechanisms. Such an interactive account may also help accommodate the context sensitivity of load effects observed across behavioral studies, including recent failures to obtain the predicted reduction in distraction under increased perceptual load (Snowden and Gray, 2025).

### 4.3 Emotional conflict and context-dependent control

Distractor congruency produced the most consistent neural effect. Incongruent trials elicited greater activity in the bilateral ACC, amygdala, DLPFC, and FEF, as well as in visual, parietal, hippocampal, striatal, and motor regions. This distributed pattern is compatible with increased perceptual, attentional, affective, and response-related demands during emotional conflict (Pessoa, 2005; Vuilleumier, 2005; Whalen et al., 2001; Wei et al., 2013; Mitchell, 2005; Shafer et al., 2012). More specifically, the striatal and motor-region involvement is consistent with increased demands on response selection and motor control during incongruent trials (Mushiake et al., 1991; Catalan et al., 1998). The involvement of visual and parietal regions further indicates that incongruency was not confined to a single conflict-control structure. The breadth of this effect is notable because behavioral congruency modulation was weak in the scanner: emotional conflict remained strongly represented neurally even when its behavioral expression was limited. At the same time, the contrast cannot distinguish perceptual mismatch from later response-selection processes, so the broad network should not be assigned to a single processing stage.

Within the left DLPFC, congruency effects depended on target emotion and cue size. Across cue size, the incongruent–congruent difference was significant for happy but not for angry targets. Decomposition of the three-way interaction further showed significant congruency effects for happy targets under two cues and angry targets under six cues. This pattern did not reproduce the behavioral happy-target reversed load effect in a one-to-one manner. Instead, it suggests that prefrontal sensitivity to emotional distractor conflict changes with the joint configuration of target emotion and cue demand, consistent with the role of lateral prefrontal systems in cognitive control over emotional distraction (Mitchell, 2005; Shafer et al., 2012).

The left ACC and right amygdala showed cue size × face size × emotion interactions, with follow-up comparisons localizing happy–angry differences to the two-cue/six-face condition in both regions. Bilateral amygdala also showed a main effect of congruency, demonstrating sensitivity to emotional target–distractor mismatch. Crucially, however, the higher-order amygdala interaction did not involve congruency and therefore cannot be interpreted as dilution-specific modulation of distractor interference. Instead, target-emotion processing in ACC and right amygdala varied with the joint cue-and-display configuration, consistent with evidence that emotional significance and attentional context interact rather than operating independently (Pessoa, 2005; Vuilleumier, 2005; Whalen et al., 2001; Shafer et al., 2012).

Target emotion also affected medial and orbital frontal regions. The primary angry-to-happy contrast engaged the left middle orbital cortex. Given the established role of orbitofrontal regions in representing and evaluating affective value, this effect may reflect differential appraisal of angry and happy expressions rather than a general threat advantage (Kringelbach and Rolls, 2004). This interpretation is intentionally cautious because the omnibus emotion effect was spatially broader than the directional contrast and because emotion-related activation can reflect multiple evaluative and control processes. Notably, target emotion did not produce a corresponding main effect in the amygdala ROIs. Because the happy and angry stimuli were selected to be comparable in arousal, this absence may be consistent with evidence that amygdala responses are strongly influenced by stimulus arousal rather than valence alone (Lin et al., 2020). Thus, matching emotional expressions on arousal may have reduced a potential source of valence-related differences in the amygdala while allowing emotion effects to emerge in other evaluative and attentional systems.

Whole-brain interactions further emphasized context dependence. In the primary analysis, the cue size × face size × distractor type interaction involved temporoparietal, striatal, and sensorimotor regions; this interaction remained significant in both robustness analyses, although its spatial extent was reduced in the equal-exposure analysis. Target emotion × distractor-type effects involved visual and motor regions; and face size × target emotion engaged the right thalamus/pulvinar, consistent with the contribution of pulvinar–cortical systems to visual attention (Zhou et al., 2016). In the primary analysis, a face size × target emotion × distractor type effect was also observed in the right middle temporal gyrus; however, this effect was not retained in the equal-exposure sensitivity analysis and should therefore be regarded as less robust. The corresponding condition-specific simple-effect contrasts for significant interactions are reported in Supplementary Table S2. These contrasts are useful for characterizing the omnibus interactions, but differences in the number or spatial extent of separately thresholded maps should not be treated as direct statistical differences between conditions. Overall, these higher-order effects indicate that the neural expression of distractor processing depended on the broader task configuration rather than reflecting distinct load- or dilution-specific pathways.

The face size × target emotion interaction further suggests that display composition altered the neural expression of target emotion rather than producing a uniform, emotion-independent effect. The omnibus interaction involved the right thalamus/pulvinar and right rolandic operculum. Under the six-face condition, emotion-related differences involved thalamic and cingulate regions in the angry > happy direction and occipital, parietal, frontal, and sensorimotor regions in the happy > angry direction, whereas under the two-face condition, happy > angry effects were more restricted to fusiform and superior temporal regions (Supplementary Table S2). This pattern suggests that increasing display density changed the neural context in which target emotion was represented, rather than uniformly enhancing or suppressing emotional processing.

### 4.4 Neural–behavioral divergence in the scanner

Robust neural congruency effects occurred despite the absence of significant behavioral modulation of congruency scores during scanning. This contrasts with Experiment 1, in which behavioral interference was greater under the larger cue condition for happy targets, indicating that the behavioral pattern observed outside the scanner was not reproduced in Experiment 2. Several factors may have contributed to this discrepancy. Scanner noise can alter cognitive control and narrow attention in Stroop-like tasks (Hommel et al., 2012). In addition, the inhibitory spillover framework proposes that sustained motor inhibition can generalize to control in unrelated domains (Berkman et al., 2009; Tuk et al., 2011). Differences in sample size may also have contributed: the smaller fMRI sample may have reduced statistical power to detect a behavioral effect, whereas the larger sample in Experiment 1 may have made the observed effect more readily detectable. Although no significant difference in BDI scores was observed between the samples (*p* > 0.05), depressive symptomatology may nevertheless represent an additional source of individual variability in behavioral performance. Procedural differences introduced by the scanner-adapted design may also have contributed. In particular, stimulus presentation was blocked in Experiment 2, rather than randomized as in Experiment 1, to maintain a stable attentional set and facilitate interpretation of the BOLD response. Previous work suggests that presentation format can influence perceptual-load effects (Theeuwes et al., 2004; Benoni et al., 2014). These explanations remain speculative; however, future studies should directly examine whether the behavioral pattern depends on scanner context, sample size, individual differences, or on whether the presentation is blocked versus randomized. The present data establish only that neural sensitivity to incongruency persisted despite weak behavioral expression during scanning. This dissociation underscores the value of combining behavioral and neural measures, while also cautioning against treating BOLD effects as direct surrogates for behavioral interference.

The additional robustness analyses also addressed the unequal amount of functional data contributed by participants. Adjusting statistically for the number of completed runs preserved the principal whole-brain pattern, including the critical Cue Size × Face Size × Distractor Type interaction. A more stringent sensitivity analysis based solely on the first two runs from each participant likewise retained seven of the nine originally significant omnibus effects and preserved a right-temporal component of the critical three-way interaction. At the same time, the face-size main effect and the face size × target emotion × distractor type interaction were not retained in the reduced-data analysis, and the spatial extent of several effects was smaller. A parallel behavioral ANCOVA further indicated that no significant association between in-scanner performance and the number of completed runs was detected (Supplementary Table S5). Together, these findings suggest that the central pattern is not solely attributable to unequal scanning exposure, while indicating that some more selective effects should be interpreted cautiously.

### 4.5 Limitations and future directions

Several limitations should guide interpretation and future work. First, the absence of significant behavioral modulation of congruency scores during fMRI limits direct behavioral–neural correspondence; scanner noise and inhibitory spillover are plausible but untested explanations. Second, the fMRI sample included only women. In Experiment 1, sex-stratified analyses showed significant cue-related effects in women, whereas no significant cue- or face-size effects emerged in men. Given the available neuroimaging resources and the primary aim of Experiment 2, this pattern informed the pragmatic decision to focus the fMRI study on a homogeneous female sample rather than divide the limited scanning budget across sex groups. The fMRI findings should therefore be interpreted as specific to women and should not be generalized to men without direct replication. Future studies should examine whether the observed neural effects generalize across sex in adequately powered mixed-sex samples. Relatedly, the fMRI sample was relatively modest (*N* = 30), reflecting the financial and practical constraints of neuroimaging data collection. This sample size may have limited sensitivity to medium-sized interaction effects, particularly higher-order interactions; future replications with larger samples would strengthen confidence in the neuroimaging findings. Third, the study used only facial stimuli, and the neutral fillers differed from emotional faces in both expression and mouth configuration. These differences may have reduced representational competition and contributed to the absence of a behavioral dilution effect. In addition, the present paradigm operationalized cue-based demand through target-location uncertainty and dilution through display composition. Testing other implementations of load and dilution will therefore be necessary to determine which aspects of the current findings generalize across selective-attention paradigms.

Another limitation is that, although depressive symptoms were used as an exclusion criterion given their documented association with attentional bias and inhibitory control (Gotlib and Joormann, 2010; Shimony et al., 2021; Yitzhak et al., 2023), anxiety was not directly assessed, despite evidence that anxiety can impair inhibitory control and efficient top-down attentional regulation and that such effects may be particularly relevant when affective distractors compete with task goals (Ansari and Derakshan, 2010; Hardin et al., 2009). Future studies should therefore incorporate a dedicated anxiety measure to evaluate its potential contribution to the present pattern of results. Finally, because continuation beyond the second functional run was not experimentally randomized and depended on participants’ willingness to continue, run number may partly reflect individual differences in fatigue tolerance, comfort, or motivation; the association between run count and whole-brain activation estimates (Supplementary Table S3) should therefore not be interpreted causally.

## 5 Conclusion

The present findings indicate that cue-based attentional demand and display composition make non-equivalent contributions to emotional distractor processing, although the supporting evidence differed in behavioral expression across experiments. In Experiment 1, increasing cue-based target uncertainty selectively enhanced behavioral interference for happy targets, whereas increasing the number of neutral faces did not reduce interference. In Experiment 2, this behavioral pattern was not replicated during scanning; nonetheless, distractor incongruency engaged a broad set of affective and attentional-control regions whose expression varied with cue size, face size, and target emotion. This behavioral–neural dissociation indicates that neural sensitivity to distraction can be evident even when its behavioral expression is weak, but it also means that the neural interactions reported here should not be interpreted as direct confirmation of the behavioral mechanism identified in Experiment 1. Taken together, these results do not support a single load- or dilution-specific neural mechanism. Instead, they favor a configuration-dependent account in which spatial uncertainty, representational competition, and emotional relevance jointly determine the processing of irrelevant information. More broadly, the study demonstrates that neural sensitivity to distraction can remain robust even when its behavioral expression is weak, underscoring the value of combining behavioral and neuroimaging measures in the study of selective attention. Because disturbances in attentional control and emotion–attention interactions are implicated across neuropsychiatric conditions, clarifying these processes in healthy cognition may also inform future mechanistic work in clinical populations.

## Data Availability

Behavioral data from Experiments 1 and 2, region-of-interest percent-signal-change (PSC) values, and associated analysis scripts are openly available on the Open Science Framework at https://osf.io/enmvy/. Raw whole-brain neuroimaging data are not publicly deposited due to file size and institutional data-sharing constraints, but are available from the corresponding author upon reasonable request.
