# Supplementary material for: Distractor processing of emotional faces: behavioral and women-only fMRI findings on the perceptual load–dilution debate
Source: Front Hum Neurosci. 2026 Sep 21;20:1963554. doi: 10.3389/fnhum.2026.1963554 (PMC13634949; doi:10.3389/fnhum.2026.1963554)
Supplement: Supplementary file 1 [file Table_1.DOCX]

**Supplementary Table S1.** Mean Reaction Times (Experiments 1 and 2)

| **Target** | **Cue** | **Face** | **Exp. 1 – Neutral** | **Exp. 1 – Congruent** | **Exp. 1 – Incongruent** | **Exp. 2 – Congruent** | **Exp. 2 – Incongruent** |
| --- | --- | --- | --- | --- | --- | --- | --- |
| Happy | 2 | 2 | 776.36 (111.38) | 771.40 (116.17) | 791.94 (116.91) | 794.15 (159.69) | 804.61 (165.96) |
| Happy | 2 | 6 | 785.40 (113.32) | 787.82 (123.52) | 792.93 (109.58) | 776.74 (147.08) | 826.30 (163.76) |
| Happy | 6 | 2 | 882.65 (113.18) | 903.29 (112.43) | 951.38 (115.41) | 933.69 (163.54) | 969.05 (166.94) |
| Happy | 6 | 6 | 1023.15 (129.87) | 1051.30 (129.98) | 1092.52 (145.09) | 1061.21 (172.77) | 1072.45 (170.55) |
| Angry | 2 | 2 | 776.49 (126.61) | 790.33 (126.25) | 795.36 (123.75) | 805.28 (144.40) | 806.10 (170.83) |
| Angry | 2 | 6 | 782.67 (113.26) | 795.71 (128.64) | 810.77 (122.78) | 807.76 (146.52) | 810.14 (141.57) |
| Angry | 6 | 2 | 883.65 (127.53) | 922.06 (134.51) | 933.35 (131.65) | 936.18 (131.22) | 958.52 (157.63) |
| Angry | 6 | 6 | 991.27 (155.67) | 1028.87 (139.71) | 1035.77 (158.53) | 1049.91 (118.69) | 1031.34 (170.66) |

Mean (SD) reaction times (ms) by target emotion, cue size, and face size across distractor conditions. Cue and Face refer to cue size and face size (2 or 6), operationalizing cue-based perceptual demand and display-based dilution, respectively. Experiment 2 did not include a neutral distractor condition.

**Supplementary Table S2.** Whole-brain T-contrast results for significant omnibus effects (Experiment 2)

| **Contrast** | **Region** | **Hemi** | **BA** | **x** | **y** | **z** | **k** | **T** |  |
| --- | --- | --- | --- | --- | --- | --- | --- | --- | --- |
| **MAIN EFFECTS** | | | | | | | | | |
| Six cues > two cues | Calcarine Sulcus (Visual Cortex) | R | BA17 | 20 | -72 | 12 | 1745 | 4.96 |  |
|  | Superior Frontal Gyrus, dorsolateral | R | BA6 | 28 | -10 | 70 | 47 | 3.97 |  |
|  | Inferior Parietal Gyrus | R | BA40 | 38 | -54 | 42 | 42 | 3.64 |  |
|  | Cerebellum, Crus I | L | – | -18 | -74 | -30 | 24 | 3.54 |  |
|  | Inferior Frontal Gyrus, opercular part | R | BA9 | 38 | 8 | 32 | 41 | 3.50 |  |
| Six faces > two faces | Paracentral Lobule | L | BA5 | -2 | -32 | 56 | 32 | 3.78 |  |
| Angry > happy | Superior Frontal Gyrus, medial | L | BA6 | -6 | 30 | 36 | 170 | 4.16 |  |
|  | Frontal Mid Orb | L | BA10 | -36 | 48 | -4 | 29 | 3.58 |  |
| Happy > angry | Cerebellum, lobule VI | L | – | -28 | -70 | -18 | 11438 | 6.40 |  |
|  | Frontal Sup Orb | R | BA47 | 18 | 30 | -10 | 39 | 4.37 |  |
|  | Inferior Parietal Gyrus | L | BA7 | -28 | -50 | 40 | 612 | 4.20 |  |
|  | Superior Occipital Gyrus | R | BA7 | 28 | -66 | 42 | 306 | 3.86 |  |
|  | Precuneus | R | – | 30 | -50 | 26 | 20 | 3.84 |  |
|  | Precentral Gyrus | L | BA6 | -38 | -8 | 58 | 142 | 3.80 |  |
|  | Middle Frontal Gyrus | R | BA6 | 38 | 4 | 52 | 69 | 3.72 |  |
| Incongruent > congruent | Calcarine Sulcus (Visual Cortex) | R | BA17 | 20 | -92 | -2 | 10155 | 7.20 |  |
|  | Hippocampus | L | BA27 | -20 | -30 | -4 | 22 | 4.31 |  |
|  | Superior Parietal Gyrus | L | BA7 | -24 | -52 | 50 | 255 | 4.08 |  |
|  | Putamen | L | – | -24 | 4 | 8 | 26 | 3.72 |  |
|  | Precentral Gyrus | R | BA6 | 44 | 0 | 24 | 31 | 3.68 |  |
| Congruent > incongruent | Precuneus | R | BA7 | 6 | -58 | 64 | 234 | 4.09 |  |
| **TWO-WAY INTERACTIONS** | | | | | | | | | |
| Six faces, angry > six faces, happy | Thalamus | L | – | -14 | -22 | 20 | 57 | 4.49 |  |
|  | Cingulum Ant | L | BA32 | -2 | 34 | 28 | 116 | 3.75 |  |
| Six faces, happy > six faces, angry | Middle Occipital Gyrus | L | BA18 | -18 | -96 | 12 | 12418 | 5.95 |  |
|  | Superior Parietal Gyrus | L | BA7 | -22 | -60 | 42 | 751 | 5.11 |  |
|  | Postcentral Gyrus | L | BA40 | -40 | -36 | 44 | 65 | 3.88 |  |
|  | Precentral Gyrus | R | BA6 | 42 | 2 | 30 | 78 | 3.82 |  |
|  | Middle Frontal Gyrus | R | BA6 | 40 | 0 | 52 | 57 | 3.58 |  |
|  | Precentral Gyrus | L | BA6 | -44 | -4 | 50 | 27 | 3.38 |  |
| Two faces, happy > two faces, angry | Fusiform Gyrus | L | BA19 | -24 | -72 | -14 | 919 | 3.86 |  |
|  | Superior Temporal Gyrus | R | BA21 | 60 | 2 | -12 | 47 | 3.72 |  |
|  | Olfactory Cortex | L | BA24 | -4 | 24 | -6 | 45 | 2.56 |  |
| Six faces, incongruent > six faces, congruent | Calcarine Sulcus (Visual Cortex) | R | BA17 | 16 | -94 | -2 | 7829 | 6.17 |  |
|  | Superior Parietal Gyrus | L | BA7 | -22 | -54 | 48 | 366 | 4.62 |  |
|  | Hippocampus | L | BA27 | -22 | -28 | -4 | 23 | 4.07 |  |
| Two faces, incongruent > two faces, congruent | Lingual Gyrus | R | BA18 | 24 | -90 | -4 | 2468 | 4.41 |  |
|  | Fusiform Gyrus | R | BA19 | 28 | -64 | -14 | 100 | 3.71 |  |
| Two faces, congruent > two faces, incongruent | Precuneus | R | BA7 | 4 | -56 | 50 | 206 | 3.72 |  |
|  | Temporal Pole, superior | L | BA22 | -50 | 14 | -4 | 48 | 3.49 |  |
| Angry, incongruent > angry, congruent | Lingual Gyrus | L | BA18 | -10 | -88 | -12 | 1558 | 4.57 |  |
|  | Fusiform Gyrus | R | BA20 | 42 | -34 | -18 | 38 | 3.95 |  |
|  | Fusiform Gyrus | R | BA19 | 30 | -60 | -12 | 74 | 3.65 |  |
| Happy, incongruent > happy, congruent | Lingual Gyrus | R | BA18 | 16 | -90 | -12 | 8866 | 6.61 |  |
|  | Globus Pallidus | L | – | -20 | -4 | 4 | 98 | 4.40 |  |
|  | Middle Frontal Gyrus | R | BA6 | 40 | -8 | 54 | 191 | 4.24 |  |
|  | Precentral Gyrus | L | BA4 | -38 | -14 | 48 | 231 | 3.94 |  |
|  | Superior Parietal Gyrus | L | BA7 | -22 | -68 | 42 | 207 | 3.84 |  |
| **THREE-WAY INTERACTIONS** | | | | | | | | | |
| Six cues, six faces: incongruent > congruent | Calcarine Sulcus (Visual Cortex) | R | BA17 | 22 | -94 | 2 | 706 | 4.71 |  |
|  | Inferior Temporal Gyrus | L | BA37 | -48 | -46 | -14 | 149 | 4.34 |  |
|  | Fusiform Gyrus | L | BA19 | -34 | -70 | -14 | 169 | 4.13 |  |
|  | Angular Gyrus | R | – | 30 | -48 | 34 | 55 | 3.94 |  |
| Six cues, six faces: congruent > incongruent | Inferior Frontal Gyrus, triangular part | R | BA9 | 30 | 26 | 26 | 31 | 3.75 |  |
|  | Middle Frontal Gyrus | L | BA9 | -34 | 50 | 36 | 42 | 3.58 |  |
| Six cues, two faces: incongruent > congruent | Lingual Gyrus | L | BA18 | -10 | -74 | -2 | 2198 | 4.92 |  |
|  | Thalamus | L | – | -4 | -26 | -8 | 69 | 3.73 |  |
|  | Lingual Gyrus | R | BA18 | 24 | -90 | -6 | 110 | 3.63 |  |
|  | Fusiform Gyrus | R | – | 30 | -64 | -16 | 23 | 3.55 |  |
| Six cues, two faces: congruent > incongruent | Supramarginal Gyrus | R | BA40 | 56 | -40 | 30 | 127 | 4.12 |  |
|  | Temporal Pole, superior | L | BA22 | -58 | 8 | -2 | 158 | 4.06 |  |
|  | Precuneus | R | BA7 | 6 | -58 | 64 | 187 | 3.92 |  |
|  | Inferior Parietal Gyrus | L | BA2 | -58 | -28 | 44 | 23 | 3.73 |  |
| Two cues, six faces: incongruent > congruent | Lingual Gyrus | R | BA18 | 20 | -88 | -12 | 4989 | 5.32 |  |
|  | Superior Occipital Gyrus | R | BA7 | 26 | -62 | 44 | 389 | 4.38 |  |
|  | Globus Pallidus | L | – | -20 | 0 | 6 | 107 | 4.15 |  |
|  | Superior Parietal Gyrus | L | BA7 | -22 | -58 | 48 | 192 | 4.11 |  |
|  | Superior Occipital Gyrus | R | BA19 | 22 | -84 | 36 | 61 | 3.81 |  |
|  | Middle Frontal Gyrus | R | BA6 | 34 | -2 | 52 | 66 | 3.63 |  |
| Two cues, six faces: congruent > incongruent | Insula | R | BA13 | 32 | 10 | 12 | 52 | 4.37 |  |
|  | Postcentral Gyrus | L | – | -36 | -18 | 32 | 51 | 3.96 |  |
|  | Precentral Gyrus | L | BA6 | -18 | -18 | 70 | 18 | 3.45 |  |
| Two cues, two faces: incongruent > congruent | Rolandic Operculum | R | BA13 | 40 | -4 | 16 | 119 | 4.25 |  |

*Peak coordinates (MNI) and cluster statistics for all significant clusters across the contrasts listed in Supplementary Table S2. All clusters survived a combined threshold of voxelwise p < .001 (uncorrected) and cluster extent k ≥ 18 voxels (corrected cluster-level α = .05; Monte Carlo cluster-extent correction, 10,000 iterations; Slotnick, 2017). Hemi = hemisphere (R = right, L = left); BA = Brodmann area (– indicates a subcortical or non-cortical region without a BA label); k = cluster size in voxels; T = peak T-value.*

**Supplementary Table S3.** Robustness comparison of whole-brain omnibus F-effects (Table 3) across three models.

| **Effect/Contrast** | **Model** | **Region** | **Hemi** | **BA** | **x** | **y** | **z** | **k** | **F** |
| --- | --- | --- | --- | --- | --- | --- | --- | --- | --- |
| Cue size | Original | Calcarine Sulcus | R | BA17 | 20 | -72 | 12 | 1324 | 24.57 |
|  |  | Superior Frontal Gyrus, dorsolateral | R | BA6 | 28 | -10 | 70 | 32 | 16.08 |
|  | Covariate | Calcarine Sulcus | R | BA17 | 20 | -72 | 12 | 1093 | 23.49 |
|  |  | Superior Frontal Gyrus, dorsolateral | R | BA6 | 28 | -10 | 70 | 22 | 15.84 |
|  | Two-run | Calcarine Sulcus | R | BA17 | 20 | -72 | 12 | 1510 | 27.93 |
|  |  | Superior Frontal Gyrus, dorsolateral | R | BA6 | 34 | -8 | 66 | 40 | 16.35 |
| Face size | Original | Paracentral Lobule | L | BA5 | -2 | -32 | 56 | 19 | 14.32 |
|  |  | Precentral Gyrus | L | BA6 | -38 | -8 | 58 | 94 | 14.93 |
|  | Covariate | Paracentral Lobule | L | BA5 | -2 | -32 | 56 | 20 | 14.40 |
|  | Two-run | Not significant |  |  |  |  |  |  |  |
| Target emotion | Original | Cerebellum | L | – | -28 | -70 | -18 | 10258 | 40.96 |
|  |  | Frontal Superior Orbital | R | BA47 | 18 | 30 | -10 | 18 | 19.11 |
|  |  | Inferior Parietal Gyrus | L | BA7 | -28 | -50 | 40 | 407 | 17.61 |
|  |  | Superior Frontal Gyrus, medial | L | BA6 | -6 | 30 | 36 | 111 | 17.31 |
|  |  | Superior Occipital Gyrus | R | BA7 | 28 | -66 | 42 | 142 | 14.92 |
|  |  | Middle Frontal Gyrus | R | BA6 | 38 | 4 | 52 | 36 | 13.36 |
|  | Covariate | Fusiform Gyrus | L | BA19 | -28 | -70 | -14 | 9474 | 36.94 |
|  |  | Middle Frontal Gyrus | L | BA10 | -36 | 48 | -4 | 26 | 14.55 |
|  |  | Superior Frontal Gyrus, medial | L | BA6 | -6 | 30 | 36 | 132 | 18.59 |
|  |  | Inferior Parietal Gyrus | L | BA7 | -28 | -50 | 40 | 332 | 17.13 |
|  |  | Superior Occipital Gyrus | R | BA7 | 28 | -66 | 42 | 71 | 13.80 |
|  |  | Precentral Gyrus | L | BA6 | -40 | -6 | 52 | 60 | 13.74 |
|  |  | Middle Frontal Gyrus | R | BA6 | 38 | 2 | 52 | 21 | 13.18 |
|  | Two-run | Lingual Gyrus | R | BA17 | 14 | -92 | -8 | 8323 | 35.98 |
|  |  | Medial Orbitofrontal Cortex | R | BA11 | 16 | 44 | -16 | 25 | 14.26 |
|  |  | Middle Occipital Gyrus | L | - | -22 | -56 | 36 | 159 | 17.75 |
|  |  | Precentral Gyrus | L | BA6 | -36 | -8 | 64 | 22 | 12.86 |
| Distractor type | Original | Calcarine Sulcus | R | BA17 | 20 | -92 | -4 | 8820 | 51.80 |
|  |  | Precuneus | R | BA7 | 6 | -58 | 64 | 175 | 16.72 |
|  |  | Superior Parietal Gyrus | L | BA7 | -24 | -52 | 50 | 131 | 16.63 |
|  |  | Superior Occipital Gyrus | R | BA39 | 28 | -60 | 36 | 79 | 15.34 |
|  | Covariate | Lingual Gyrus | R | BA18 | 22 | -90 | -6 | 8241 | 47.90 |
|  |  | Precentral Gyrus | R | BA6 | 44 | 0 | 24 | 18 | 13.68 |
|  |  | Superior Parietal Gyrus | L | BA7 | -24 | -52 | 50 | 122 | 15.78 |
|  |  | Precuneus | R | BA7 | 6 | -58 | 64 | 191 | 16.85 |
|  | Two-run | Lingual Gyrus | R | BA18 | 22 | -88 | -8 | 7424 | 50.87 |
|  |  | Inferior Frontal Gyrus, triangular part | R | BA47 | 54 | 22 | -2 | 21 | 13.67 |
|  |  | Superior Temporal Gyrus | L | BA22 | -60 | -46 | 12 | 23 | 14.85 |
|  |  | Superior Occipital Gyrus | R | BA31 | 26 | -72 | 16 | 18 | 13.24 |
|  |  | Inferior Parietal Gyrus | L | BA7 | -28 | -56 | 46 | 54 | 13.40 |
|  |  | Precuneus | R | BA7 | 2 | -48 | 60 | 18 | 12.34 |
| Face × Emotion | Original | Thalamus | R | – | 4 | -26 | 10 | 27 | 16.27 |
|  |  | Rolandic Operculum | R | BA43 | 54 | -16 | 20 | 40 | 14.29 |
|  | Covariate | Thalamus | R | - | 4 | -26 | 10 | 25 | 16.45 |
|  |  | Rolandic Operculum | R | BA43 | 54 | -16 | 20 | 50 | 14.64 |
|  | Two-run | Thalamus | R | - | 4 | -28 | 12 | 21 | 13.99 |
| Face × Type | Original | Precuneus | R | BA7 | 18 | -58 | 44 | 49 | 13.72 |
|  | Covariate | Precuneus | R | BA7 | 18 | -58 | 44 | 46 | 13.99 |
|  | Two-run | Superior Occipital Gyrus | R | BA19 | 18 | -84 | 36 | 24 | 15.00 |
|  |  | Precuneus | R | BA7 | 12 | -60 | 46 | 69 | 15.20 |
| Emotion × Type | Original | Precentral Gyrus | R | BA4 | 42 | -14 | 56 | 132 | 18.95 |
|  |  | Lingual Gyrus | R | BA30 | 8 | -60 | 8 | 200 | 15.49 |
|  | Covariate | Lingual Gyrus | R | BA30 | 8 | -60 | 8 | 130 | 14.34 |
|  |  | Precentral Gyrus | R | BA4 | 44 | -14 | 52 | 119 | 18.02 |
|  | Two-run | Lingual Gyrus | R | BA30 | 4 | -66 | 6 | 258 | 14.68 |
|  |  | Precentral Gyrus | R | BA4 | 42 | -14 | 56 | 74 | 16.48 |
| Face × Emotion × Type | Original | Middle Temporal Gyrus | R | BA21 | 62 | -8 | -14 | 24 | 13.97 |
|  | Covariate | Middle Temporal Gyrus | R | BA21 | 62 | -8 | -14 | 22 | 13.66 |
|  | Two-run | Not significant |  |  |  |  |  |  |  |
| Cue × Face × Type | Original | Paracentral Lobule | R | BA6 | 12 | -31 | 58 | 26 | 18.44 |
|  |  | Superior Temporal Gyrus | R | BA22 | 62 | -50 | 20 | 178 | 17.79 |
|  |  | Putamen | R | – | 32 | 8 | 10 | 44 | 17.51 |
|  |  | Supramarginal Gyrus | L | BA40 | -64 | -28 | 28 | 55 | 15.20 |
|  |  | Precentral Gyrus | R | BA6 | 50 | -2 | 26 | 42 | 13.27 |
|  | Covariate | Putamen | R | – | 32 | 8 | 10 | 52 | 18.74 |
|  |  | Superior Temporal Gyrus | R | BA22 | 62 | -50 | 20 | 208 | 18.89 |
|  |  | Precentral Gyrus | R | BA6 | 50 | -2 | 26 | 55 | 13.29 |
|  |  | Supramarginal Gyrus | L | BA40 | -64 | -28 | 28 | 77 | 16.12 |
|  |  | Paracentral Lobule | R | BA4 | 12 | -32 | 58 | 30 | 18.96 |
|  | Two-run | Middle Temporal Gyrus | R | BA22 | 58 | -52 | 18 | 37 | 14.43 |

All clusters shown met the nominal threshold of voxelwise p < .001 (uncorrected) and cluster extent k ≥ 18 voxels. “Not significant” indicates that the effect did not meet this threshold in that model. For the robustness models (covariate-adjusted and two-run), k ≥ 18 was retained as a fixed threshold for nominal comparison with the primary analysis and does not represent a newly recomputed Monte Carlo cluster-extent correction for either model. BA, Brodmann area (–, subcortical/non-cortical); Hemi, hemisphere; k, cluster size in voxels; F, peak F-value.

**Supplementary Table S4.** Robustness comparison of whole-brain directional T-contrasts (Table 4) across three models.

| **Effect/Contrast** | **Model** | **Region** | **Hemi** | **BA** | **x** | **y** | **z** | **k** | **T** |
| --- | --- | --- | --- | --- | --- | --- | --- | --- | --- |
| 6-cue > 2-cue | Original | Calcarine Sulcus | R | BA17 | 20 | -72 | 12 | 1745 | 4.96 |
|  |  | Superior Frontal Gyrus, dorsolateral | R | BA6 | 28 | -10 | 70 | 47 | 3.97 |
|  |  | Inferior Parietal Gyrus | R | BA40 | 38 | -54 | 42 | 42 | 3.64 |
|  |  | Cerebellum, Crus I | L | – | -18 | -74 | -30 | 24 | 3.54 |
|  |  | Inferior Frontal Gyrus, opercular part | R | BA9 | 38 | 8 | 32 | 41 | 3.50 |
|  | Covariate | Calcarine Sulcus | R | BA17 | 20 | -72 | 12 | 1473 | 4.85 |
|  |  | Inferior Frontal Gyrus, opercular part | R | BA9 | 38 | 8 | 32 | 22 | 3.36 |
|  |  | Inferior Parietal Gyrus | R | BA40 | 38 | -54 | 42 | 29 | 3.54 |
|  |  | Superior Frontal Gyrus, dorsolateral | R | BA6 | 28 | -10 | 70 | 39 | 3.98 |
|  | Two-run | Fusiform Gyrus | L | BA19 | -22 | -76 | -12 | 29 | 3.34 |
|  |  | Calcarine Sulcus | R | BA17 | 20 | -72 | 12 | 2067 | 5.28 |
|  |  | Thalamus | M | - | 0 | -28 | 4 | 20 | 3.48 |
|  |  | Inferior Frontal Gyrus, triangular part | R | BA9 | 40 | 24 | 24 | 32 | 3.58 |
|  |  | Superior Frontal Gyrus, dorsolateral | R | BA6 | 34 | -8 | 66 | 55 | 4.04 |
| 6-face > 2-face | Original | Paracentral Lobule | L | BA5 | -2 | -32 | 56 | 32 | 3.78 |
|  | Covariate | Paracentral Lobule | L | BA5 | -2 | -32 | 56 | 43 | 3.80 |
|  | Two-run | Not significant |  |  |  |  |  |  |  |
| Angry > happy | Original | Superior Frontal Gyrus, medial | L | BA6 | -6 | 30 | 36 | 170 | 4.16 |
|  |  | Frontal Mid Orbital | L | BA10 | -36 | 48 | -4 | 29 | 3.58 |
|  | Covariate | Middle Frontal Gyrus | L | BA10 | -36 | 48 | -4 | 53 | 3.81 |
|  | Covariate | Superior Frontal Gyrus, medial | L | BA6 | -6 | 30 | 36 | 189 | 4.31 |
|  | Two-run | Superior Frontal Gyrus, medial | L | BA6 | -6 | 34 | 34 | 29 | 3.43 |
| Incongruent > congruent | Original | Calcarine Sulcus | R | BA17 | 20 | -92 | -2 | 10155 | 7.20 |
|  |  | Hippocampus | L | BA27 | -20 | -30 | -4 | 22 | 4.31 |
|  |  | Superior Parietal Gyrus | L | BA7 | -24 | -52 | 50 | 255 | 4.08 |
|  |  | Putamen | L | – | -24 | 4 | 8 | 26 | 3.72 |
|  |  | Precentral Gyrus | R | BA6 | 44 | 0 | 24 | 31 | 3.68 |
|  | Covariate | Lingual Gyrus | R | BA18 | 22 | -90 | -6 | 9430 | 6.92 |
|  | Covariate | Hippocampus | L | BA27 | -20 | -30 | -4 | 18 | 4.19 |
|  | Covariate | Putamen | L | - | -24 | 4 | 8 | 24 | 3.75 |
|  | Covariate | Precentral Gyrus | R | BA6 | 44 | 0 | 24 | 36 | 3.70 |
|  | Covariate | Superior Parietal Gyrus | L | BA7 | -24 | -52 | 50 | 244 | 3.97 |
|  | Two-run | Lingual Gyrus | R | BA18 | 22 | -88 | -8 | 8308 | 7.13 |
|  | Two-run | Inferior Parietal Gyrus | L | BA7 | -28 | -56 | 46 | 94 | 3.66 |
| 2-cue 2-face: incongruent > congruent | Original | Rolandic Operculum | R | BA13 | 40 | -4 | 16 | 119 | 4.25 |
|  | Covariate | Rolandic Operculum | R | BA13 | 40 | -4 | 16 | 110 | 4.23 |
|  | Two-run | Lingual Gyrus | R | BA18 | 22 | -90 | -6 | 40 | 3.48 |
| 2-cue 6-face: incongruent > congruent | Original | Lingual Gyrus | R | BA18 | 20 | -88 | -12 | 4989 | 5.32 |
|  |  | Superior Occipital Gyrus | R | BA7 | 26 | -62 | 44 | 389 | 4.38 |
|  |  | Globus Pallidus | L | – | -20 | 0 | 6 | 107 | 4.15 |
|  |  | Superior Parietal Gyrus | L | BA7 | -22 | -58 | 48 | 192 | 4.11 |
|  |  | Superior Occipital Gyrus | R | BA19 | 22 | -84 | 36 | 61 | 3.81 |
|  |  | Middle Frontal Gyrus | R | BA6 | 34 | -2 | 52 | 66 | 3.63 |
|  | Covariate | Lingual Gyrus | R | BA18 | 20 | -88 | -12 | 4319 | 5.14 |
|  |  | Globus Pallidus | L | - | -20 | 0 | 6 | 97 | 4.07 |
|  |  | Superior Occipital Gyrus | R | BA19 | 22 | -86 | 36 | 67 | 3.77 |
|  |  | Superior Occipital Gyrus | R | BA7 | 26 | -62 | 44 | 357 | 4.25 |
|  |  | Superior Parietal Gyrus | L | BA7 | -22 | -58 | 48 | 163 | 4.02 |
|  |  | Middle Frontal Gyrus | R | BA6 | 34 | -2 | 52 | 62 | 3.60 |
|  | Two-run | Lingual Gyrus | R | BA18 | 20 | -88 | -14 | 3947 | 5.11 |
|  |  | Superior Occipital Gyrus | R | BA19 | 22 | -86 | 36 | 72 | 3.73 |
|  |  | Superior Occipital Gyrus | R | BA7 | 26 | -64 | 44 | 278 | 4.38 |
|  |  | Superior Parietal Gyrus | L | BA7 | -22 | -58 | 48 | 70 | 3.55 |
|  |  | Precentral Gyrus | R | BA6 | 30 | -8 | 52 | 52 | 3.70 |
| 6-cue 2-face: incongruent > congruent | Original | Lingual Gyrus | L | BA18 | -10 | -74 | -2 | 2198 | 4.92 |
|  |  | Thalamus | L | – | -4 | -26 | -8 | 69 | 3.73 |
|  |  | Lingual Gyrus | R | BA18 | 24 | -90 | -6 | 110 | 3.63 |
|  |  | Fusiform Gyrus | R | – | 30 | -64 | -16 | 23 | 3.55 |
|  | Covariate | Lingual Gyrus | L | BA18 | -10 | -74 | -2 | 1501 | 4.48 |
|  |  | Thalamus | L | - | -4 | -26 | -8 | 47 | 3.59 |
|  | Two-run | Fusiform Gyrus | L | BA19 | -22 | -80 | -18 | 2271 | 4.52 |
| 6-cue 6-face: incongruent > congruent | Original | Calcarine Sulcus | R | BA17 | 22 | -94 | 2 | 706 | 4.71 |
|  |  | Inferior Temporal Gyrus | L | BA37 | -48 | -46 | -14 | 149 | 4.34 |
|  |  | Fusiform Gyrus | L | BA19 | -34 | -70 | -14 | 169 | 4.13 |
|  |  | Angular Gyrus | R | – | 30 | -48 | 34 | 55 | 3.94 |
|  | Covariate | Fusiform Gyrus | L | BA19 | -34 | -70 | -14 | 221 | 4.32 |
|  |  | Inferior Temporal Gyrus | L | BA37 | -48 | -46 | -14 | 177 | 4.50 |
|  |  | Superior Occipital Gyrus | R | BA17 | 20 | -94 | 4 | 864 | 4.73 |
|  |  | Angular Gyrus | R | - | 30 | -48 | 34 | 117 | 4.14 |
|  | Two-run | Fusiform Gyrus | L | BA19 | -34 | -70 | -14 | 213 | 4.15 |
|  |  | Inferior Temporal Gyrus | L | BA37 | -48 | -46 | -14 | 178 | 4.28 |
|  |  | Calcarine Sulcus | R | BA17 | 18 | -92 | 2 | 586 | 4.33 |
| 2-face: incongruent > congruent | Original | Lingual Gyrus | R | BA18 | 24 | -90 | -4 | 2468 | 4.41 |
|  |  | Fusiform Gyrus | R | BA19 | 28 | -64 | -14 | 100 | 3.71 |
|  | Covariate | Lingual Gyrus | R | BA18 | 22 | -90 | -6 | 1532 | 4.10 |
|  |  | Fusiform Gyrus | R | BA19 | 28 | -64 | -14 | 43 | 3.42 |
|  | Two-run | Lingual Gyrus | R | BA18 | 22 | -90 | -6 | 1581 | 5.11 |
| 6-face: incongruent > congruent | Original | Calcarine Sulcus | R | BA17 | 16 | -94 | -2 | 7829 | 6.17 |
|  |  | Superior Parietal Gyrus | L | BA7 | -22 | -54 | 48 | 366 | 4.62 |
|  |  | Hippocampus | L | BA27 | -22 | -28 | -4 | 23 | 4.07 |
|  | Covariate | Calcarine Sulcus | R | BA17 | 16 | -94 | -2 | 7764 | 6.09 |
|  |  | Hippocampus | L | BA27 | -22 | -28 | -4 | 29 | 4.12 |
|  |  | Superior Parietal Gyrus | L | BA7 | -22 | -56 | 48 | 407 | 4.64 |
|  |  | Precentral Gyrus | R | BA6 | 32 | 0 | 50 | 19 | 3.34 |
|  | Two-run | Calcarine Sulcus | R | BA17 | 16 | -94 | 2 | 6122 | 5.85 |
|  |  | Superior Occipital Gyrus | R | BA7 | 24 | -58 | 38 | 175 | 3.98 |
|  |  | Superior Parietal Gyrus | L | BA7 | -24 | -56 | 50 | 282 | 4.47 |
| Angry: incongruent > congruent | Original | Lingual Gyrus | L | BA18 | -10 | -88 | -12 | 1558 | 4.57 |
|  |  | Fusiform Gyrus | R | BA20 | 42 | -34 | -18 | 38 | 3.95 |
|  |  | Fusiform Gyrus | R | BA19 | 30 | -60 | -12 | 74 | 3.65 |
|  | Covariate | Fusiform Gyrus | R | BA20 | 42 | -34 | -18 | 23 | 3.79 |
|  |  | Lingual Gyrus | L | BA18 | -10 | -88 | -10 | 739 | 4.16 |
|  |  | Fusiform Gyrus | R | BA19 | 30 | -60 | -12 | 20 | 3.35 |
|  |  | Calcarine Sulcus | R | BA17 | 18 | -92 | 0 | 228 | 3.95 |
|  | Two-run | Fusiform Gyrus | L | BA19 | -26 | -74 | -12 | 1075 | 4.61 |
|  |  | Lingual Gyrus | R | BA18 | 22 | -90 | -6 | 415 | 4.43 |
|  |  | Fusiform Gyrus | R | BA19 | 30 | -66 | -12 | 42 | 3.45 |
| Happy: incongruent > congruent | Original | Lingual Gyrus | R | BA18 | 16 | -90 | -12 | 8866 | 6.61 |
|  |  | Globus Pallidus | L | – | -20 | -4 | 4 | 98 | 4.40 |
|  |  | Middle Frontal Gyrus | R | BA6 | 40 | -8 | 54 | 191 | 4.24 |
|  |  | Precentral Gyrus | L | BA4 | -38 | -14 | 48 | 231 | 3.94 |
|  |  | Superior Parietal Gyrus | L | BA7 | -22 | -68 | 42 | 207 | 3.84 |
|  | Covariate | Lingual Gyrus | R | BA18 | 16 | -90 | -12 | 9026 | 6.71 |
|  |  | Globus Pallidus | L | - | -20 | -4 | 4 | 138 | 4.57 |
|  |  | Superior Parietal Gyrus | L | BA7 | -22 | -68 | 42 | 294 | 4.07 |
|  |  | Precentral Gyrus | L | BA6 | -36 | -10 | 50 | 315 | 4.08 |
|  |  | Middle Frontal Gyrus | R | BA6 | 40 | -8 | 54 | 265 | 4.41 |
|  |  | Supplementary Motor Area | R | BA6 | 8 | 8 | 50 | 30 | 3.53 |
|  | Two-run | Lingual Gyrus | R | BA18 | 16 | -90 | -12 | 7878 | 6.97 |
|  |  | Globus Pallidus | L | - | -20 | -4 | 4 | 26 | 3.63 |
|  |  | Precentral Gyrus | L | BA4 | -36 | -12 | 52 | 224 | 3.93 |
|  |  | Precentral Gyrus | R | BA4 | 40 | -12 | 52 | 72 | 3.81 |
| 2-face angry: incongruent > congruent | Original | Calcarine Sulcus | R | BA17 | 12 | -64 | 8 | 1198 | 4.33 |
|  |  | Precentral Gyrus | L | BA4 | -36 | -12 | 52 | 152 | 3.79 |
|  |  | Superior Temporal Gyrus | R | BA22 | 70 | -22 | 0 | 45 | 3.78 |
|  |  | Cerebellum, Crus I | L | – | -20 | -80 | -20 | 126 | 3.63 |
|  |  | Lingual Gyrus | R | BA18 | 14 | -88 | -14 | 49 | 3.58 |
|  |  | Precentral Gyrus | R | BA4 | 40 | -12 | 50 | 37 | 3.57 |
|  | Covariate | Inferior Frontal Gyrus, triangular part | L | BA10 | -38 | 40 | 0 | 69 | 3.80 |
|  |  | Superior Frontal Gyrus, medial | L | BA6 | -8 | 28 | 36 | 29 | 3.72 |
|  | Two-run | Lingual Gyrus | R | BA18 | 22 | -90 | -6 | 121 | 4.11 |
| 2-face happy: incongruent > congruent | Original | Superior Frontal Gyrus, medial | L | BA6 | -8 | 28 | 36 | 54 | 3.96 |
|  |  | Inferior Frontal Gyrus, triangular part | L | BA10 | -38 | 40 | 0 | 124 | 3.94 |
|  |  | Fusiform Gyrus | R | BA19 | 30 | -60 | -8 | 56 | 3.72 |
|  |  | Frontal Superior Orbital | L | BA10 | -34 | 60 | -2 | 20 | 3.62 |
|  |  | Lingual Gyrus | R | BA18 | 22 | -90 | -6 | 35 | 3.49 |
|  | Covariate | Cerebellum, Crus I | L | – | -20 | -80 | -20 | 84 | 3.52 |
|  |  | Lingual Gyrus | R | BA18 | 14 | -88 | -14 | 38 | 3.52 |
|  |  | Calcarine Sulcus | R | BA30 | 12 | -64 | 8 | 1162 | 4.35 |
|  |  | Superior Temporal Gyrus | R | BA22 | 70 | -22 | 0 | 68 | 3.93 |
|  |  | Precentral Gyrus | R | BA4 | 40 | -12 | 50 | 51 | 3.68 |
|  |  | Precentral Gyrus | L | BA4 | -36 | -12 | 50 | 170 | 3.81 |
|  | Two-run | Cerebellum, Crus I | L | – | -18 | -84 | -20 | 1294 | 4.20 |
|  |  | Precentral Gyrus | R | BA4 | 40 | -12 | 50 | 44 | 3.74 |
|  |  | Precentral Gyrus | L | BA4 | -36 | -12 | 52 | 136 | 3.71 |
| 6-face angry: incongruent > congruent | Original | Lingual Gyrus | R | BA18 | 22 | -88 | -10 | 8272 | 6.02 |
|  |  | Superior Parietal Gyrus | L | BA7 | -22 | -56 | 46 | 409 | 4.25 |
|  |  | Middle Frontal Gyrus | R | BA6 | 36 | 0 | 54 | 93 | 3.74 |
|  | Covariate | Calcarine Sulcus | L | BA17 | -10 | -90 | -10 | 177 | 3.77 |
|  |  | Thalamus | L | - | -10 | -24 | 6 | 18 | 3.61 |
|  | Two-run | Lingual Gyrus | L | BA18 | -12 | -88 | -12 | 221 | 3.63 |
| 6-face happy: incongruent > congruent | Original | Calcarine Sulcus | L | BA18 | -10 | -90 | -10 | 328 | 3.93 |
|  |  | Thalamus | L | – | -10 | -24 | 6 | 25 | 3.83 |
|  | Covariate | Lingual Gyrus | R | BA18 | 22 | -88 | -10 | 8977 | 6.30 |
|  |  | Superior Parietal Gyrus | L | BA7 | -20 | -62 | 46 | 541 | 4.46 |
|  |  | Middle Frontal Gyrus | R | BA6 | 36 | 0 | 54 | 155 | 3.96 |
|  | Two-run | Lingual Gyrus | R | BA18 | 16 | -90 | -12 | 7198 | 5.93 |
|  |  | Superior Parietal Gyrus | L | BA7 | -24 | -56 | 50 | 167 | 4.03 |

All clusters shown met the nominal threshold of voxelwise p < .001 (uncorrected) and cluster extent k ≥ 18 voxels. “Not significant” indicates that the effect did not meet this threshold in that model. For the robustness models (covariate-adjusted and two-run), k ≥ 18 was retained as a fixed threshold for nominal comparison with the primary analysis and does not represent a newly recomputed Monte Carlo cluster-extent correction for either model. BA, Brodmann area (–, subcortical/non-cortical); Hemi, hemisphere (R = right, L = left, M = midline); k, cluster size in voxels; T, peak T-value.

**Supplementary Table S5.** Behavioral robustness analysis for Experiment 2 including run count as a covariate.

| **Source** | **df** | **F** | **p** | **η²_p_** |
| --- | --- | --- | --- | --- |
| Run count (main effect) | 1, 28 | 0.06 | .813 | .002 |
| Cue size × Run count | 1, 28 | 0.23 | .635 | .008 |
| Face size × Run count | 1, 28 | 1.60 | .217 | .054 |
| Target emotion × Run count | 1, 28 | 0.15 | .704 | .005 |
| Cue size × Face size × Run count | 1, 28 | 0.23 | .633 | .008 |
| Cue size × Target emotion × Run count | 1, 28 | 0.15 | .700 | .005 |
| Face size × Target emotion × Run count | 1, 28 | 2.24 | .145 | .074 |
| Cue size × Face size × Target emotion × Run count | 1, 28 | 0.47 | .498 | .017 |

*N* = 30. Only the run-count main effect and its interactions with the within-subjects factors are shown; the primary within-subjects effects (cue size, face size, target emotion, and their interactions) are reported in the main text. No significant association between run count and in-scanner behavioral performance was detected, and no interaction involving run count reached significance.
